# Addressing confounding artifacts in reconstruction of gene co-expression networks

**DOI:** 10.1186/s13059-019-1700-9

**Published:** 2019-05-16

**Authors:** Princy Parsana, Claire Ruberman, Andrew E. Jaffe, Michael C. Schatz, Alexis Battle, Jeffrey T. Leek

**Affiliations:** 1 Department of Computer Science, Johns Hopkins University, Baltimore, MD USA; 2 Department of Biostatistics, Johns Hopkins Bloomberg School of Public Health, Baltimore, MD USA; 3 Lieber Institute for Brain Development, Johns Hopkins Medical Campus, Baltimore, MD USA; 4 Department of Mental Health, Johns Hopkins Bloomberg School of Public Health, Baltimore, MD USA; 5 McKusick-Nathans Institute of Genetic Medicine, Johns Hopkins University School of Medicine, Baltimore, MD USA; 6 Center for Computational Biology, Johns Hopkins University, Baltimore, MD USA; 7 Department of Biology, Johns Hopkins University, Baltimore, MD USA; 8 Department of Biomedical Engineering, Johns Hopkins University, Baltimore, MD USA

## Abstract

Gene co-expression networks capture biological relationships between genes and are important tools in predicting gene function and understanding disease mechanisms. We show that technical and biological artifacts in gene expression data confound commonly used network reconstruction algorithms. We demonstrate theoretically, in simulation, and empirically, that principal component correction of gene expression measurements prior to network inference can reduce false discoveries. Using data from the GTEx project in multiple tissues, we show that this approach reduces false discoveries beyond correcting only for known confounders.

**Electronic supplementary material:**

The online version of this article (10.1186/s13059-019-1700-9) contains supplementary material, which is available to authorized users.

## Background

Gene co-expression networks seek to identify transcriptional patterns indicative of functional interactions and regulatory relationships between genes [1–3]. These are not yet fully characterized for most species, tissues, and disease-relevant contexts. Therefore, reconstructing co-expression networks from high-throughput measurements is of common interest. However, accurate reconstruction of such networks remains a challenging problem.

Though some specialized methods for the reconstruction of co-expression networks do consider confounding signals within their model [4, 5], routinely used network learning methods [6, 7] do not directly account for technical and unwanted biological effects known to confound gene expression data. Despite this, many studies do not employ any form of data correction or correct only for known confounders prior to network reconstruction (Additional file 1: Table S1**)**. These artifacts influence gene expression measurements, often introducing spurious correlations between genes [8–10]. These correlations are often inferred as relationships between genes, leading to inaccurate network structure and erroneous conclusions in downstream analyses [4, 5, 8, 11, 12]. Therefore, it is critical to correct gene expression data for unwanted biological and technical variation without eliminating signal of interest before applying standard network learning methods.

## Results and discussion

In this study, we provide a framework for data correction leveraging the structure of scale-free networks. We show that for scale-free networks, principal components of a gene expression matrix can consistently identify components that reflect artifacts in the data rather than network relationships. It has been shown that real-world networks including co-expression networks often have scale-free topology, i.e., the node degree distribution of these networks follow a power law [13–15]. Several studies have employed the assumption of scale-free topology to infer high-dimensional gene co-expression and splicing networks [6, 16].

**Fig. 1:**
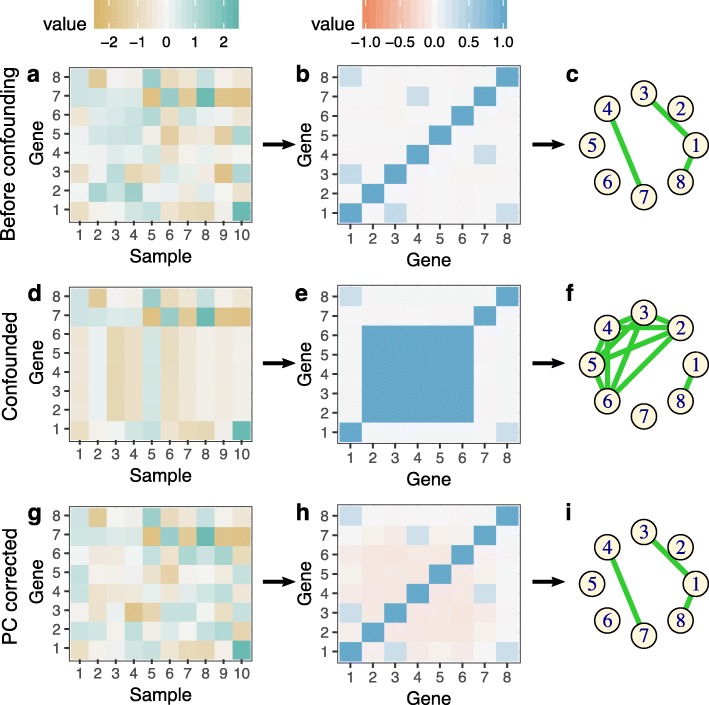
Toy simulation example. (**a-f**) This toy simulation shows the reconstruction of gene co-expression networks is affected by confounders. (**g-i**) True underlying network structure can be reconstructed after principal component correction of gene expression data as described in the paper

Latent factor-based data correction has been successfully employed in many applications in genomics from genome-wide association studies, cis- and trans-eQTL mapping, to differential expression analysis [9, 17–20]. In genome-wide association studies investigating the association between genotype and complex traits, it has been shown that top principal components explain the broad correlation between genotypes which generally reflects population structure rather than a desired functional biological signal of interest [20]. Co-expression analysis is more complicated because confounders affect sets of genes in ways that resemble co-expression. Here, we show mathematically, through simulation (Fig. 1, Additional file 1: Notes 1, 2.1, and 2.2; Additional files 2 and 3) and through real data examples that similar to genetic association studies, the broad correlation between gene expression levels in uncorrected data appears to reflect artifacts. We expect that most real co-expression networks are sparse which means that most genes are only connected to a small subset of other genes. We prove that when such networks satisfy the scale-free property, the signals from the network will not be sufficiently broad across genes to influence the latent variable estimates from PCA. Thus, principal components will primarily capture latent confounders, which can then be regressed from the expression data before network reconstruction is performed (Additional file 1: Note 1).

Using a toy and scale-free simulation, we first showed that confounding can introduce false correlations between sets of genes that can mimic co-expression and can lead to false edge discovery during reconstruction of co-expression networks with graphical lasso—sometimes at the expense of losing true connections (Fig. 1d-f, Additional file 2). We corrected the confounded simulated data using our PC-based approach and reconstructed the network using the residuals. Graphical lasso correctly estimated the network structure obtained from corrected data, which was the same as the true network structure that was obtained from the original simulated data (Fig. 1a-c,g-h, Additional file 2). We also simulated multivariate Gaussian data with 350 samples and 5000 genes from an underlying scale-free network (Additional file 3). Similar to the previous simulation, we found that confounding in data can introduce a lot more false positives in reconstructed co-expression networks. We also showed that networks reconstructed with PC corrected data in this setting were more similar to original simulated data compared to confounded data (Additional file 3). Throughout our analysis, to estimate the number of principal components to be removed, we used a permutation-based scheme [21] as implemented in the sva package [22].

To demonstrate the impact of latent confounders and principal component correction on the reconstruction of co-expression networks from real large-scale human gene expression measurements, we applied our method to RNA-Seq data from the Genotype-Tissue Expression (GTEx) project v6p release. We considered data from eight diverse tissues containing between 304 and 430 samples each (Additional file 1: Table S2): Subcutaneous adipose, lung, skeletal muscle, thyroid, whole blood, tibial artery, tibial nerve, and sun-exposed skin. Using the most variable 5000 genes (Additional file 1: Notes 2 and 4), we reconstructed co-expression networks for each tissue with two popular methods: (a) weighted gene co-expression network analysis [6, 23] and (b) graphical lasso [7, 24]. Since the true underlying co-expression network structure is not known, we assessed the networks using gene pairs annotated to function in the same pathways [25, 26] as ground truth edges.

We inferred networks obtained by using (a) uncorrected expression data, the residuals after regressing out (b) RNA integrity number (RIN), (c) exonic rate—a mapping covariate that corresponds to fraction of reads mapped to exons, (d) sample-specific estimate of GC bias, all known to be common confounders in mRNA gene expression data [27–29], and (e) residuals from multiple regression model using covariates that explained at least 1% of expression variance (adjusted *R*^2^ ≥ 0.01, Additional file 1: Table S3–S5) [28, 30–33].

**Fig. 2:**
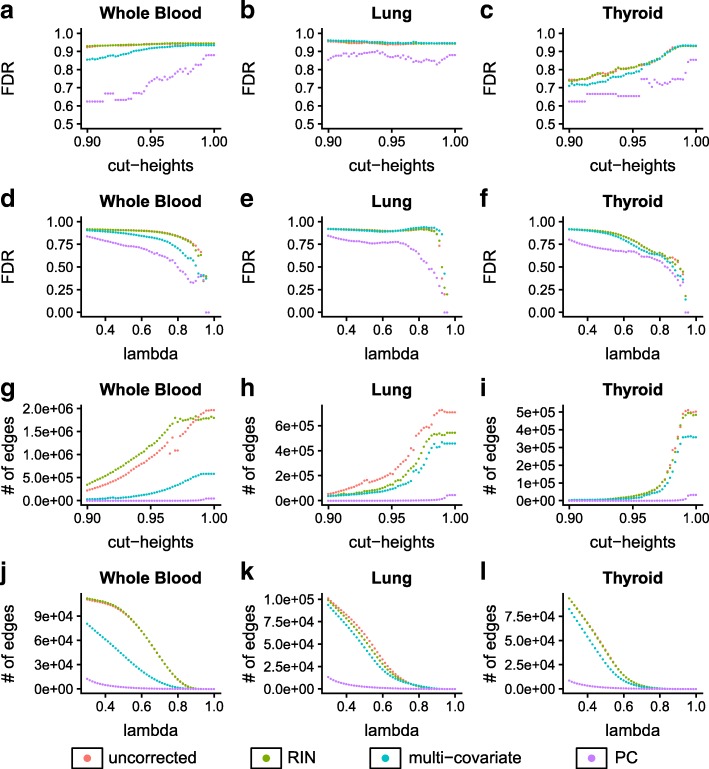
False discovery rate of WGCNA modules and graphical lasso networks based on canonical pathways (**a**–**f**). The density of networks inferred from PC-corrected data is sparser (**g**–**l**). **a**–**c** FDR of WGCNA networks obtained at varying cut heights. Each point corresponds to FDR of the network obtained at a specific cut height. Each color represents networks reconstructed with a specific correction approach. **d**–f Each point in the figure corresponds to false discovery rates of networks obtained at a specific L1 penalty parameter value (lambda) in the graphical lasso. Each color represents networks reconstructed with a specific correction approach—uncorrected, multi-covariate, RIN, and PC corrected. **g**–**i** Each point corresponds to a number of edges in networks inferred by WGCNA at a cut height. **j**–**l** Each point corresponds to a number of edges inferred by graphical lasso in networks obtained at a specific L1 penalty parameter value. Networks inferred by PC-corrected data have fewer edges compared to uncorrected or RIN-corrected data

Co-expression gene modules obtained from weighted signed co-expression networks (Additional file 1: Note 2.4) were interpreted as fully connected subgraphs, as is standard. For most tissues, networks obtained from data corrected for latent confounders showed fewer false discoveries compared to those obtained from uncorrected data or from correcting for individual covariates including RIN, exonic rate (a quality metric from RNA-Seq mapping), or sample-specific GC bias (Fig. 2, Additional file 1: Figures S1, S3, and S8). Improved performance of networks obtained from PC corrected data was more evident in the whole blood, skeletal muscle, tibial artery, tibial nerve, subcutaneous adipose, and thyroid. But for some tissues such as the lung, PC correction only contributes to moderate improvement on false discovery rates in the reconstructed networks. It is possible that in these cases, the networks may violate the scale-free assumption or that true signal was already sufficiently strong in the raw data. We also observed that correcting gene expression data with multiple technical covariates (approximately 9–17 were used per tissue, Additional file 1: Table S5) sometimes improved the reconstruction of co-expression networks obtained by WGCNA (Fig. 2a–c, Additional file 1: Figure S1). Average WGCNA module size for networks with cut-height greater than 0.99 was smaller with PC-corrected data compared to uncorrected counterparts (Additional file 1: Figure S15). We also observed that the number of genes assigned to the gray (unassigned) module in WGCNA was considerably higher in PC-corrected networks (Additional file 1: Figure S15). Finally, we repeated this analysis by varying multiple settings of WGCNA and found that PC corrected showed improvement in most tissues consistently (Additional file 1: Figures S10 and S11).

In graphical lasso networks, we found that networks estimated with principal component corrected data showed fewer false discoveries compared to networks estimated with uncorrected, RIN-corrected or multiple covariates corrected data (Fig. 2d–f, Additional file 1: Figure S2). We observed that in generally improved performance on false discoveries in PC corrected networks over raw data in the whole blood, the skeletal muscle, tibial artery, and tibial nerve. Compared to raw data, jointly correcting the gene expression data for multiple technical covariates that affect expression measurements also improved reconstruction with graphical lasso in some tissues such as the whole blood, thyroid, and tibial artery, while it showed little to no improvement over uncorrected data in the lung, muscle, tibial nerve, and sun-exposed skin (Fig. 2d–f**,** Additional file 1: Figure S2). However, we observed that across all tissues, PC correction still shows fewer false discoveries compared to multiple technical covariate-based correction. There was no visible improvement in network reconstruction between using uncorrected data and residuals from RIN or exonic rate, thereby suggesting that RIN, exonic rate, or GC bias individually is not a sufficient alternative for the wide range of confounding variation found in gene expression data (Fig. 2, Additional file 1: Figures S2, S4, and S9). We also found that there was no improvement on false negative rates upon PC correction in networks built with WGCNA or graphical lasso (Additional file 1: Figure S14).

With both WGCNA and graphical lasso, networks inferred from principal component corrected data were much sparser than networks from uncorrected and RIN, exonic rate, or GC bias corrected counterparts (Fig. 2g–l). Further, PC corrected networks from graphical lasso also showed higher clustering coefficient and fewer hubs compared to others (Additional file 1: Figures S12 and S13).

## Conclusion

Network reconstruction methods are vulnerable to latent confounders present in gene expression data. Co-expression networks obtained from data corrected for effects of RIN, exonic rate, or GC bias individually show little improvement on false discoveries compared to uncorrected data and are not a sufficient surrogate for the diverse sources of confounding variation in gene expression data. With empirical analysis supported by theoretical proof, we show that PC correction is a simple, yet effective approach to address confounding variation for the reconstruction of gene co-expression networks. We do note for particularly dense or connected sub-graphs in the underlying biological system that may not match the scale-free assumption, or when large differences in expression changes are expected (e.g., cancer vs normal), removing principal components may remove biological signal of interest and, as with any data cleaning methodology, should be used with caution. We have implemented our PC correction approach as a function—“sva_network” in *sva* Bioconductor package which can be used prior to network reconstruction with a range of methods (Vignette: Additional file 4).

## Methods

### Principal component-based correction of gene expression

Using a permutation-based approach as described in [21], we first determined the number of principal components “*p*” to correct the data for with the “num.sv” function in the Bioconductor package *sva* (Additional file 1: Table S4). Next, we compute the principal component loadings *L* of the standardized expression matrix with singular value decomposition (SVD). Using a linear model, we regressed the top “*p*” principal components (*p* as determined by “num.sv”) on each gene *E*_*i*_ from the expression data and computed the residuals $\widehat{E_i}$.

$$ {E}_i={\mu}_i+{\beta}_i\times {L}_{1:p} $$

$$ {\widehat{E}}_i={E}_i-\left[{\mu}_i+\left({\beta}_i\times {L}_{1:p}\right)\right] $$

### Evaluation of co-expression networks

To evaluate our correction method and its effect on the reconstruction of co-expression networks, we used two methods to infer the structure of gene co-expression networks: (a) weighted gene co-expression networks (WGCNA) [10] and (b) graphical lasso [11] (Additional file 1: Note 2).

Since the underlying network structure is generally unknown, we used genes known to be functional in the same pathways as ground truth to assess these networks.

Any pair of genes that have at least one pathway in common were assumed as a true functional relationship. An edge that was observed between a pair of genes in the inferred network (from WGCNA or graphical lasso) and was also present in the list of real connections was called as a true positive (TP). We defined false positive (FP) to be an edge that was observed between a pair of genes in the inferred network, however was absent in the list of real connections.

- Shared true positives: We obtained a refined list of real connections described above by restricting to pairs of genes that were present in at least two pathway databases.

All TP, FP, and FN were computed with genes restricted to the most variable 5000 genes that were used for reconstructing co-expression networks. We compute the false discovery rate as given below:

$$ \mathrm{FDR}=\frac{\mathrm{FP}}{\mathrm{TP}+\mathrm{FP}} $$

## Additional files

Additional file 1: Supplemental methods and results. This file contains theoretical proofs, supplemental methods, results, figures, and tables. (PDF 963 kb)

Additional file 2: Scale-free simulation (R notebook) (HTML 772 kb)

Additional file 3: Scale-free simulation with sample and gene numbers matched to GTEx (R notebook). (HTML 763 kb)

Additional file 4: Tutorial vignette to apply PC correction prior to network reconstruction in an example dataset. (HTML 726 kb)

## Abbreviations

WGCNA: Weighted gene co-expression networks
