# Supplementary material for: Addressing confounding artifacts in reconstruction of gene co-expression networks
Source: Genome Biol. 2019 May 16;20:94. doi: 10.1186/s13059-019-1700-9 (PMC6521369; doi:10.1186/s13059-019-1700-9)
Supplement: Supplementary file 1 — Supplemental methods and results. This file contains theoretical proofs, supplemental methods, results, figures, and tables. (PDF 963 kb) [file 13059_2019_1700_MOESM1_ESM.pdf]

Supplementary Information

Supplementary Note 1: Conditions for and Proof of Convergence of Principal Components

Lemma: *Let* $\mathbf{X}$ *be a high-dimensional matrix of expression data with signal both due to artifacts* $\mathbf{A}$*, and due to a genuine network of linear expression relationships. Then under the conditions below and provided that the node degree distribution of the network follows a power-law, the principal components of* X *consistently estimate a linear space spanning the artifacts* A *and not the network structure.*

Proof:
Decompose a gene expression matrix with n samples and m genes $\mathbf{X}_{m\times n} = (\mathbf{x}_1, ..., \mathbf{x}_m)^T$ as follows:

$$\mathbf{X} = \boldsymbol{\mu} \times \mathbf{1} + \boldsymbol{\Gamma}_\mathbf{A}\mathbf{A} + \boldsymbol{\Gamma}_\mathbf{N}\mathbf{N} + \mathbf{U}$$

where,

- $\boldsymbol{\mu} = (\mu_1, ..., \mu_m)^T$ is an m dimensional column vector with $\mu_i := E[\mathbf{x}_i]$, $i = 1, ..., m$ and $\mathbf{1}$ is an n dimensional row vector of 1's.
- There are L artifacts or confounders ($L < n$), forming an $L \times n$ matrix A with an associated coefficient matrix Γ_A.
- N is an $m \times n$ matrix of expression data without any network structure, with associated $m \times m$ coefficient vector Γ_N. Features i and k are share an edge if γ^N_{ik} or γ^N_{ki} are nonzero. This represents a linear relationship between the expression levels of genes. To avoid circularity, the diagonal entries of Γ_N are set to zero.
- $\mathbf{U}$ is an $m \times n$ matrix of pairwise independent mean zero random noise

Based on our previous work [Leek, 2011], given a high-dimensional matrix with the number of features much larger than the number of samples ($m >> n$) we make the following additional assumptions about the behavior of the data in the experiment.

1. The number of non-zero entries in the network Γ_N follows a power-law distribution with an exponential coefficient $2 < \alpha < 3$ [Barabási et al., 2000]. As we point out in the main text power-law degree distributions have been observed in gene expression networks, for example yeast co-expression networks [Van Noort et al., 2004, Carlson et al., 2006] and *Caenorhabditis elegans* [Kim et al., 2001], and the preferential attachment model characteristic of scale-free networks has been explained by gene duplication [Rzhetsky and Gomez, 2001, Bhan et al., 2002, Jordan et al., 2004]. Further, network inference algorithms such as WGCNA also employ this assumption.

2. The entries in the artifact and network coefficient, pre-network expression data, and independent noise matrices have bounded fourth moment:

$$0 < E\left[\left(\gamma_{A_{i,j}}\right)^4\right] \leq B_{\gamma_A}$$
$$0 < E\left[\left(\gamma_{N_{i,j}}\right)^4\right] \leq B_{\gamma_N}$$
$$0 < E\left[(N_{i,j})^4\right] \leq B_N$$
$$0 < E\left[(u_{i,j})^4\right] \leq B_U.$$

   Therefore, by Lyapunov's inequality, there exist (finite) bounds B'_{γ_A}, B'_{γ_N}, B'_N, and B'_U, on the variances:

$$0 < \text{Var}\left(\gamma_{A_{i,j}}\right) = E\left[(\gamma_{A_{i,j}})^2\right] \leq B'_{\gamma_A}$$
$$0 < \text{Var}\left(\gamma_{N_{i,j}}\right) = E\left[(\gamma_{N_{i,j}})^2\right] \leq B'_{\gamma_N}$$
$$0 < \text{Var}\left(N_{i,j}\right) = E\left[(N_{i,j})^2\right] \leq B'_N$$
$$0 < \text{Var}\left(u_{i,j}\right) = E\left[(u_{i,j})^2\right] \leq B'_U.$$

   This is true for most common distributions used to model gene expression data or a suitably transformed version.

3. There exists a positive definite matrix Δ for which the following hold:
   (a) $\lim_{m\to\infty} \|\frac{1}{m}A^T\Gamma_A^T\Gamma_A A - A^T\Delta A\|_F = 0$
   (b) $A^T\Delta A$ has eigenvalues $\lambda_1 > .... > \lambda_L > \lambda_{L+1} = ... = \lambda_n = 0$

   This assumption means that the batch effects and other artifacts are sufficiently widespread as to affect a fixed and non-negligable percentage of the genes in the data set.

Additionally, we assume without loss of generality, that expression levels of each gene in $\mathbf{X}$ is centered.

4. $\boldsymbol{\mu} = \vec{0}$.

5. The expression data in the absence of any network structure, N, has mean $E[N] = \vec{0}$ where $\vec{0}$ is an m-dimensional column vector. Further, in the absence of network structure, the genes are pairwise independent. Therefore, by Assumption 2 the entries of N converge almost surely to zero.

Based on this model, we show that the principal components of the matrix $\mathbf{X}$ (with a fixed n - sample size) estimate the artifacts and are not corrupted by the signal from the network terms.
The eigen-vectors of the matrix $\frac{1}{m}\mathbf{X}^T\mathbf{X}$ are equal to the right singular vectors of the matrix $\mathbf{X}$. Given

observed data $\mathbf{X}$, the empirical variance-covariance matrix of the data $\hat{\Sigma}$ takes the form:

$$\begin{aligned}
\hat{\Sigma} &= \frac{1}{m}\mathbf{X}^T\mathbf{X} \\
&= \frac{1}{m}\left(\Gamma_A A + \Gamma_N N + \mathbf{U}\right)^T\left(\Gamma_A A + \Gamma_N N + \mathbf{U}\right) \\
&= \frac{1}{m}\left(A^T\Gamma_A^T + N^T\Gamma_N^T + \mathbf{U}^T\right)\left(\Gamma_A A + \Gamma_N N + \mathbf{U}\right) \\
&= \frac{1}{m}(A^T\Gamma_A^T\Gamma_A A + A^T\Gamma_A^T\Gamma_N N + A^T\Gamma_A^T\mathbf{U} + N^T\Gamma_N^T\Gamma_A A + N^T\Gamma_N^T\Gamma_N N + N^T\Gamma_N^T\mathbf{U} + \\
&\quad \mathbf{U}^T\Gamma_A A + \mathbf{U}^T\Gamma_N N + \mathbf{U}^T\mathbf{U}) \\
&= \frac{1}{m}\left(A^T\Gamma_A^T\Gamma_A A + A^T\Gamma_A^T\Gamma_N N + N^T\Gamma_N^T\Gamma_A A + N^T\Gamma_N^T\Gamma_N N\right) + \\
&\quad \frac{1}{m}\left(A^T\Gamma_A^T\mathbf{U} + N^T\Gamma_N^T\mathbf{U} + \mathbf{U}^T\Gamma_A A + \mathbf{U}^T\Gamma_N N + \mathbf{U}^T\mathbf{U}\right) \\
&= \frac{1}{m}A^T\Gamma_A^T\Gamma_A A + \frac{1}{m}A^T\Gamma_A^T\Gamma_N N + \frac{1}{m}N^T\Gamma_N^T\Gamma_A A + \frac{1}{m}N^T\Gamma_N^T\Gamma_N N + \\
&\quad \frac{1}{m}A^T\Gamma_A^T\mathbf{U} + \frac{1}{m}N^T\Gamma_N^T\mathbf{U} + \frac{1}{m}\mathbf{U}^T\Gamma_A A + \frac{1}{m}\mathbf{U}^T\Gamma_N N + \frac{1}{m}\mathbf{U}^T\mathbf{U}
\end{aligned}$$

We will show that as the number of features (i.e. genes) grows, the empirical variance-covariance matrix, after centering by an estimate of the background variation, converges to the same thing as if there were no network structure:

$$\tilde{\mathbf{X}}_{unstr} := \Gamma_A A + \mathbf{U}.$$

Then we can show that the principal components of the confounded matrix are consistent estimators of the confounding variables.
Therefore, we will show that, holding the number of observations n fixed, there exits an $n \times n$ matrix $\mathcal{L}$ so that:

$$\lim_{m\to\infty}\frac{1}{m}\left(\tilde{\mathbf{X}}^{\text{unstr}}\right)^T\tilde{\mathbf{X}}^{\text{unstr}} - \hat{\sigma}^2_{ave}\mathbf{I} = \mathcal{L}$$

$$\lim_{m\to\infty}\frac{1}{m}\mathbf{X}^T\mathbf{X} - \hat{\sigma}^2_{ave}\mathbf{I} = \mathcal{L}$$

where, borrowing the notation from Leek 2011, we let $V_L(\mathbf{X}) = \{v_1(\mathbf{X}), ..., v_L(\mathbf{X})\}$ be a matrix of the first L right singular vectors of $\mathbf{X}$ and $\hat{\Gamma}_L$ the least squares estimates from regressing $\mathbf{X}$ on $V_L(\mathbf{X})$. Then, we define:

$$\sigma^2_{ave} := \frac{1}{m(n-L)}\|\mathbf{X} - \hat{\Gamma}_L V_L(\mathbf{X})\|_F,$$

where we estimate L using a permutation approach through the 'num.sv' function in the *sva* package.

To determine $\mathcal{L}$, we write:

$$\begin{aligned}
\frac{1}{m}\left(\tilde{\mathbf{X}}^{\text{unstr}}\right)^T \tilde{\mathbf{X}}^{\text{unstr}} - \hat{\sigma}^2_{ave}\mathbf{I} &= \frac{1}{m}\left(\Gamma_A A + U\right)^T \left(\Gamma_A A + U\right) - \hat{\sigma}^2_{ave}\mathbf{I} \\
&= \frac{1}{m}\left(A^T \Gamma_A^T + U^T\right)\left(\Gamma_A A + U\right) - \hat{\sigma}^2_{ave}\mathbf{I} \\
&= \frac{1}{m}A^T\Gamma_A^T\Gamma_A A + \frac{1}{m}A^T\Gamma_A^T U + \frac{1}{m}U^T\Gamma_A A + \frac{1}{m}{}^T U - \hat{\sigma}^2_{ave}\mathbf{I}
\end{aligned}$$

Letting m (number of genes) grow,

$$\begin{aligned}
&\lim_{m\to\infty}\frac{1}{m}\left(\tilde{\mathbf{X}}^{\text{unstr}}\right)^T \tilde{\mathbf{X}}^{\text{unstr}} - \hat{\sigma}^2_{ave}\mathbf{I} \\
&\quad= \lim_{m\to\infty}\frac{1}{m}\Gamma_A^T\Gamma_A A + \lim_{m\to\infty}\frac{1}{m}A^T\Gamma_A^T U + \lim_{m\to\infty}\frac{1}{m}U^T\Gamma_A A + \lim_{m\to\infty}\frac{1}{m}U^T U - \hat{\sigma}^2_{ave}\mathbf{I} \\
&\quad= A^T\Delta A + \lim_{m\to\infty}\frac{1}{m}A^T\Gamma_A^T U + \lim_{m\to\infty}\frac{1}{m}U^T\Gamma_A A + \lim_{m\to\infty}\frac{1}{m}U^T U - \hat{\sigma}^2_{ave}\mathbf{I}
\end{aligned}$$

Leek 2011 shows that the terms $\lim_{m\to\infty}\frac{1}{m}A^T\Gamma_A^T U + \lim_{m\to\infty}\frac{1}{m}U^T\Gamma_A A$ both converge almost surely to zero by the Kolmogorov Strong Law of Large Numbers (KSLLN). Further, Leek 2011 uses KSLLN to show that the off diagonal elements of $\frac{1}{m}U^T U$ converge almost surely to zero, while the diagonals converge almost surely to $\hat{\sigma}^2_{ave}$. Therefore,

$$\lim_{m\to\infty}\frac{1}{m}\left(\tilde{\mathbf{X}}^{\text{unstr}}\right)^T \tilde{\mathbf{X}}^{\text{unstr}} - \hat{\sigma}^2_{ave}\mathbf{I} = A^T\Delta A,$$

and

$$\mathcal{L} = A^T\Delta A.$$

The limit of the empirical variance-covariance matrix is as follows:

$$\lim_{m\to\infty}\frac{1}{m}\mathbf{X}^T\mathbf{X}-\hat{\sigma}^2_{ave}\mathbf{I}$$
$$=\lim_{m\to\infty}\frac{1}{m}A^T\Gamma_A^T\Gamma_A A+\lim_{m\to\infty}\frac{1}{m}\Gamma_A A^T\Gamma_N N+\lim_{m\to\infty}\frac{1}{m}N^T\Gamma_N^T\Gamma_A A+\lim_{m\to\infty}\frac{1}{m}N^T\Gamma_N^T\Gamma_N N+-\hat{\sigma}^2_{ave}\mathbf{I}$$
$$\lim_{m\to\infty}\frac{1}{m}A^T\Gamma_A^T\mathbf{U}+\lim_{m\to\infty}\frac{1}{m}N^T\Gamma_N^T\mathbf{U}+\lim_{m\to\infty}\frac{1}{m}\mathbf{U}^T\Gamma_A A+\lim_{m\to\infty}\frac{1}{m}\mathbf{U}^T\Gamma_N N+\lim_{m\to\infty}\frac{1}{m}\mathbf{U}^T\mathbf{U}-\hat{\sigma}^2_{ave}\mathbf{I}$$
$$=\lim_{m\to\infty}\frac{1}{m}A^T\Gamma_A^T\Gamma_A A+\lim_{m\to\infty}\frac{1}{m}\Gamma_A A^T U+\lim_{m\to\infty}\frac{1}{m}U^T\Gamma_A A+\lim_{m\to\infty}\frac{1}{m}U^T U-\hat{\sigma}^2_{ave}\mathbf{I}+$$
$$\lim_{m\to\infty}\frac{1}{m}A^T\Gamma_A^T\Gamma_N N+\lim_{m\to\infty}\frac{1}{m}N^T\Gamma_N^T\Gamma_A A+\lim_{m\to\infty}\frac{1}{m}N^T\Gamma_N^T\Gamma_N N+\lim_{m\to\infty}\frac{1}{m}\mathbf{U}^T\Gamma_N N+\lim_{m\to\infty}\frac{1}{m}N^T\Gamma_N^T\mathbf{U}$$
$$=A^T\Delta A+\lim_{m\to\infty}\frac{1}{m}U^T U-\hat{\sigma}^2_{ave}\mathbf{I}+\lim_{m\to\infty}\frac{1}{m}A^T\Gamma_A^T\Gamma_N N+\lim_{m\to\infty}\frac{1}{m}N^T\Gamma_N^T\Gamma_A A+$$
$$\lim_{m\to\infty}\frac{1}{m}N^T\Gamma_N^T\Gamma_N N+\lim_{m\to\infty}\frac{1}{m}\mathbf{U}^T\Gamma_N N+\lim_{m\to\infty}\frac{1}{m}N^T\Gamma_N^T\mathbf{U}$$
$$=A^T\Delta A+\underbrace{\lim_{m\to\infty}\frac{1}{m}A^T\Gamma_A^T\Gamma_N N}_{(1)}+\underbrace{\lim_{m\to\infty}\frac{1}{m}N^T\Gamma_N^T\Gamma_A A}_{(2)}+\underbrace{\lim_{m\to\infty}\frac{1}{m}N^T\Gamma_N^T\Gamma_N N}_{(3)}+$$
$$\underbrace{\lim_{m\to\infty}\frac{1}{m}\mathbf{U}^T\Gamma_N N}_{(4)}+\underbrace{\lim_{m\to\infty}\frac{1}{m}N^T\Gamma_N^T\mathbf{U}}_{(5)}$$

We consider the convergence of (1) through (5) separately:

1.
$$\lim_{m\to\infty}\frac{1}{m}A^T\Gamma_A^T\Gamma_N N=\lim_{m\to\infty}A^T\frac{1}{m}\Gamma_A^T\Gamma_N N$$

We first consider $Q:=\frac{1}{m}\Gamma_A^T\Gamma_N$, an $L\times m$ matrix with entries indexed by $l\in\{1,...,L\},k\in\{1,...,m\}$:

$$\begin{aligned}q_{lk}&=Q_{l,k}\\&=\frac{1}{m}\sum_{j=1}^{m}\Gamma_{A_{j,l}}\Gamma_{N_{j,k}}\\&=\frac{1}{m}\sum_{j=1}^{m}\gamma_{A_{j,l}}\gamma_{N_{j,k}}\\&=\frac{1}{m}\sum_{\{j:\gamma_{N_{j,k}}\neq 0\}}\gamma_{A_{j,l}}\gamma_{N_{j,k}}+\frac{1}{m}\sum_{\{j:\gamma_{N_{j,k}}=0\}}\gamma_{A_{j,l}}\gamma_{N_{j,k}}\\&=\frac{1}{m}\sum_{\{j:\gamma_{N_{j,k}}\neq 0\}}\gamma_{A_{j,l}}\gamma_{N_{j,k}}+\frac{1}{m}\sum_{\{j:\gamma_{N_{j,k}}=0\}}\gamma_{A_{j,l}}\times 0\\&=\frac{1}{m}\sum_{\{j:\gamma_{N_{j,k}}\neq 0\}}\gamma_{A_{j,l}}\gamma_{N_{j,k}}\end{aligned}$$

Suppose that there are $0 \leq d \leq m$ indices j for which $\gamma_{N_{j,k}} \neq 0$, so that there are d terms $\gamma_{A_{j,l}}\gamma_{N_{j,k}}$ in the summation contributing to q_{lk}. We can re-index these terms as $\gamma_{A_{j',l}}\gamma_{N_{j',k}}, j' = 1, ..., d$.

For any fixed k, whenever $\gamma_{N_{j,k}}$, necessarily genes k and j share an edge. Therefore, given d non-zero coefficients $\gamma_{N_{j,k}}$, gene k has at least degree d. However, [Newman, 2003] show that for scale free networks following a power-law degree distribution $p_k \sim k^{\alpha-1}$, as assumed in our framework, the maximum degree of a vertex in the network follows $k_{\max} \sim m^{\frac{1}{\alpha-1}}$, and $d \leq m^{\frac{1}{\alpha-1}}$. Therefore, we can write each element as:

$$
\begin{aligned}
q_{lk} &= \frac{1}{m} \sum_{\{j:\gamma_{N_{j,k}} \neq 0\}} \gamma_{A_{j,l}}\gamma_{N_{j,k}} \\
&= \frac{1}{m} \sum_{j'=1}^{d} \gamma_{A_{j',l}}\gamma_{N_{j',k}} \\
&= \frac{d}{m}\frac{1}{d} \sum_{j'=1}^{d} \gamma_{A_{j',l}}\gamma_{N_{j',k}} \\
&\leq \frac{m^{\frac{1}{\alpha-1}}}{m}\frac{1}{d} \sum_{j'=1}^{d} \gamma_{A_{j',l}}\gamma_{N_{j',k}} \\
&= m^{-1}m^{\frac{1}{\alpha-1}}\frac{1}{d} \sum_{j'=1}^{d} \gamma_{A_{j',l}}\gamma_{N_{j',k}} \\
&= m^{\frac{2-\alpha}{\alpha-1}}\frac{1}{d} \sum_{j'=1}^{d} \gamma_{A_{j',l}}\gamma_{N_{j',k}} \\
&= m^{\frac{-(\alpha-2)}{\alpha-1}}\frac{1}{d} \sum_{j'=1}^{d} \gamma_{A_{j',l}}\gamma_{N_{j',k}} \\
&= \frac{1}{m^{\frac{\alpha-1}{\alpha-2}}}\frac{1}{d} \sum_{j'=1}^{d} \gamma_{A_{j',l}}\gamma_{N_{j',k}}
\end{aligned}
$$

By Assumption 1 ($2 < \alpha < 3$), so that $\frac{\alpha-1}{\alpha-2} > 1$ and $\lim_{m\to\infty} \frac{1}{m^{\frac{\alpha-1}{\alpha-2}}} = 0$.

Now, consider the expectation of the terms inside of the summation. For any j', applying the Cauchy-Schwarz inequality to $|\gamma_{A_{j',l}}|$ and $|\gamma_{N_{j',k}}|$

$$
\begin{aligned}
E\left[|\gamma_{A_{j',l}}\gamma_{N_{j',k}}|\right] &\leq \sqrt{E\left[|\gamma_{A_{j',l}}|^2\right]E\left[|\gamma_{N_{j',k}}|^2\right]} \\
&= \sqrt{E\left[(\gamma_{A_{j',l}})^2\right]E\left[(\gamma_{N_{j',k}})^2\right]} \\
&\leq \sqrt{B'_{\gamma_A} \times B'_{\gamma_N}} \quad \text{By Assumption 2} \\
&= B^* \quad \text{where we define the bound } B^* := \sqrt{B'_{\gamma_A} \times B'_{\gamma_N}},
\end{aligned}
$$

and

$$
-\infty < -B^* \leq E\left[\gamma_{A_{j',l}}\gamma_{N_{j',k}}\right] \leq B^* < \infty,
$$

and by the Strong Law of Large Numbers,

$$\frac{1}{d}\sum_{j'=1}^{d}\gamma_{A_{j',l}}\gamma_{N_{j',k}}\xrightarrow{a.s.}E\left[\gamma_{A_{j',l}}\gamma_{N_{j',k}}\right],$$

therefore, for each l,k:

$$q_{lk}\leq\frac{1}{m^{\frac{\alpha-1}{\alpha-2}}}\frac{1}{d}\sum_{j'=1}^{d}\gamma_{A_{j',l}}\gamma_{N_{j',k}}\xrightarrow{a.s.}0$$

s

and

$$Q\xrightarrow{a.s.}0.$$

Recall, the matrix of artifacts A is $L\times n$ dimensional, so that it is fixed with respect to m, and, as shown in Assumption 5, $N\xrightarrow{a.s.}0$, so that by Slutsky's Theorem:

$$\lim_{m\to\infty}\frac{1}{m}A^T\Gamma_A^T\Gamma_N N=0$$

2.

$$\lim_{m\to\infty}\frac{1}{m}N^T\Gamma_N^T\Gamma_A A$$

By symmetry, the same argument as in (1) holds, and

$$\lim_{m\to\infty}\frac{1}{m}N^T\Gamma_N^T\Gamma_A A=0$$

3.

$$\lim_{m\to\infty}\frac{1}{m}N^T\Gamma_N^T\Gamma_N N=\lim_{m\to\infty}N^T\frac{1}{m}\Gamma_N^T\Gamma_N N$$

We will first consider $P:=\frac{1}{m}\Gamma_N^T\Gamma_N$, an $m\times m$ matrix with entries indexed by $l,k\in\{1,...,m\}$:

$$\begin{aligned}p_{lk}&=P_{l,k}\\&=\frac{1}{m}\sum_{j=1}^{m}\Gamma_{N_{j,l}}\Gamma_{N_{j,k}}\\&=\frac{1}{m}\sum_{j=1}^{m}\gamma_{N_{j,l}}\gamma_{N_{j,k}}\end{aligned}$$

We will consider the diagonal and off-diagonal entries of P separately. The diagonal entries

$(k = l)$ take the form:

$$\begin{aligned} p_{ll} &= \frac{1}{m}\sum_{j=1}^{m}\gamma_{N_{j,l}}\gamma_{N\,j,l} \\ &= \frac{1}{m}\sum_{j=1}^{m}\gamma_{N_{j,l}}^2 \\ &= \frac{1}{m}\sum_{\{j:\gamma_{N_{j,l}}\neq 0\}}\gamma_{N_{j,l}}^2 + \frac{1}{m}\sum_{\{j:\gamma_{N_{j,l}}=0\}}\gamma_{N_{j,l}}^2 \\ &= \frac{1}{m}\sum_{\{j:\gamma_{N_{j,l}}\neq 0\}}\gamma_{N_{j,l}}^2 \end{aligned}$$

Now, whenever $\gamma_{N_{j,l}} \neq 0$, by definition, genes j and l share an edge, so that d', the number of j such that $\gamma_{N_{j,l}} \neq 0$ is equal to the degree of vertex l. Following the argument from the proof of (1) , $d' \leq m^{\frac{1}{\alpha-1}}, 2 < \alpha < 3$ and:

$$\begin{aligned} p_{ll} &= \frac{1}{m}\sum_{\{j:\gamma_{N_{j,l}}\neq 0\}}\gamma_{N_{j,l}}^2 \\ &= \frac{1}{m}\sum_{j'=1}^{d'}\gamma_{N_{j',l}}^2 \\ &= \frac{d'}{m}\frac{1}{d'}\sum_{j'=1}^{d'}\gamma_{N_{j',l}}^2 \\ &\leq \frac{1}{m^{\frac{\alpha-1}{\alpha-2}}}\frac{1}{d'}\sum_{j'=1}^{d'}\gamma_{N_{j',l}}^2 \end{aligned}$$

Again, by Assumption 1

$$\lim_{m\to\infty}\frac{1}{m^{\frac{\alpha-1}{\alpha-2}}} = 0.$$

Further, by Assumption 2

$$E\left[\gamma_{N_{j',l}}^4\right] \leq B_{\gamma_N},$$

so that applying the Strong Law of Large Numbers,

$$\frac{1}{d}\sum_{j'=1}^{d'}\gamma_{N_{j',l}}^2 \xrightarrow{a.s.} E\left[\gamma_{N_{j',l}}^2\right] \leq B'_{\gamma_N},$$

and for each l:

$$0 \leq p_{ll} \leq \frac{1}{m^{\frac{\alpha-1}{\alpha-2}}}\frac{1}{d'}\sum_{j'=1}^{d'}\gamma_{N_{j',l}}^2 \xrightarrow{a.s.} 0.$$

We now consider the off-diagonal entries($k \neq l$):

$$\begin{aligned}
p_{lk} &= P_{l,k} \\
&= \frac{1}{m}\sum_{j=1}^{m} \Gamma_{N_{j,l}}\Gamma_{N_{j,k}} \\
&= \frac{1}{m}\sum_{j=1}^{m} \gamma_{N_{j,l}}\gamma_{N_{j,k}} \\
&= \frac{1}{m}\sum_{\{j:\gamma_{N_{j,l}}\neq 0 \text{ and } \gamma_{\mathrm{N_{j,k}}}\neq 0\}} \gamma_{N_{j,l}}\gamma_{N_{j,k}} + \frac{1}{m}\sum_{\{j:\gamma_{N_{j,l}}=0 \text{ or } \gamma_{\mathrm{N_{j,k}}}=0\}} \gamma_{N_{j,l}}\gamma_{N_{j,k}} \\
&= \frac{1}{m}\sum_{\{j:\gamma_{N_{j,l}}\neq 0 \text{ and } \gamma_{\mathrm{N_{j,k}}}\neq 0\}} \gamma_{N_{j,l}}\gamma_{N_{j,k}}
\end{aligned}$$

If both $\gamma_{N_{j,l}} \neq 0$ and $\gamma_{N_{j,k}} \neq 0$ then gene j shares and edge with both genes l and k, so that d', the number of j such that $\gamma_{N_{j,l}} \neq 0$ and $\gamma_{N_{j,k}} \neq 0$ will be bounded by the maximum of the degrees of vertices l and k. The same argument as used for the diagonal entries then follows:

$$p_{lk} \leq \frac{1}{m^{\frac{\alpha-1}{\alpha-2}}}\frac{1}{d''}\sum_{j'=1}^{d'} \gamma_{N_{j',l}}\gamma_{N_{j',k}},$$

and

$$\lim_{m\to\infty} \frac{1}{m^{\frac{\alpha-1}{\alpha-2}}} = 0.$$

Further, for any j', by Assumption 2 and the Cauchy-Schwarz inequality to $|\gamma_{N_{j',l}}|$ and $|\gamma_{N_{j',k}}|$

$$\begin{aligned}
E\left[|\gamma_{N_{j',l}}\gamma_{N_{j',k}}|\right] &\leq \sqrt{E\left[|\gamma_{N_{j',l}}|^2\right] E\left[|\gamma_{N_{j',k}}|^2\right]} \\
&= \sqrt{E\left[(\gamma_{N_{j',l}})^2\right] E\left[(\gamma_{N_{j',k}})^2\right]} \\
&\leq \sqrt{B'_{\gamma_N} \times B'_{\gamma_N}}
\end{aligned}$$

and

$$-\infty < -(B'_{\gamma_N})^2 \leq E\left[\gamma_{N_{j',l}}\gamma_{N_{j',k}}\right] (B'_{\gamma_N})^2 < \infty,$$

and by the Strong Law of Large Numbers,

$$\frac{1}{d''}\sum_{j'=1}^{d''} \gamma_{N_{j',l}}\gamma_{N_{j',k}} \xrightarrow{a.s.} E\left[\gamma_{N_{j',l}}\gamma_{N_{j',k}}\right],$$

therefore, for each $l \neq k$:

$$p_{lk} \leq \frac{1}{m^{\frac{\alpha-1}{\alpha-2}}}\frac{1}{d''}\sum_{j'=1}^{d''} \gamma_{N_{j',l}}\gamma_{N_{j',k}} \xrightarrow{a.s.} 0.$$

Therefore, both the diagonal and off-diagonal entries in P converge to zero, and

$$P \xrightarrow{a.s.} 0.$$

As shown in Assumption 5, $N \xrightarrow{a.s.} 0$, so that by Slutsky's Theorem:

$$\lim_{m\to\infty} \frac{1}{m} N^T \Gamma_N^T \Gamma_N N = 0$$

4.
$$\lim_{m\to\infty} \frac{1}{m}\mathbf{U}^T\Gamma_N N$$

This term converges almost surely to zero by the KSLLN since $E[U] = 0$ and Γ_N and U have bounded fourth moments.

5.
$$\lim_{m\to\infty} \frac{1}{m}N^T\Gamma_N^T\mathbf{U}$$

This term converges almost surely to zero by the KSLLN since $E[U] = 0$ and Γ_N and U have bounded fourth moments.

Therefore, all of the terms (1)-(5) converge almost surely to zero and the limit of the empirical variance-covariance matrix is

$$\lim_{m\to\infty} \frac{1}{m}\mathbf{X}^T\mathbf{X} - \hat{\sigma}^2_{ave}\mathbf{I} = \mathbf{A^T}\boldsymbol{\Delta}\mathbf{A} + \underbrace{\lim_{m\to\infty}\frac{1}{m}A^T\Gamma_A^T\Gamma_N N}_{(1)} + \underbrace{\lim_{m\to\infty}\frac{1}{m}N^T\Gamma_N^T\Gamma_A A}_{(2)} +$$
$$\underbrace{\lim_{m\to\infty}\frac{1}{m}N^T\Gamma_N^T\Gamma_N N}_{(3)} + \underbrace{\lim_{m\to\infty}\frac{1}{m}\mathbf{U}^T\Gamma_N N}_{(4)} + \underbrace{\lim_{m\to\infty}\frac{1}{m}N^T\Gamma_N^T\mathbf{U}}_{(5)} = A^T\Delta A = \mathcal{L}$$

The principal components of this matrix consistently estimate the space spanned by the confounding artifacts as we have previously demonstrated [Leek, 2011].
Therefore we show that given confounded high-dimensional gene expression data where the number of genes is much larger than the number of samples - top principal components will consistently estimate artifacts, and not network structure.

Supplementary Note 2: Supplementary Methods

All analyses was performed using R and scripts are available on github at:
`https://github.com/leekgroup/networks_correction`

2.1 Toy simulation example

We construct a true underlying network with eight nodes that represent genes and three edges that represent conditional dependencies between the genes. Next, we simulate 10,000 observations from a multivariate normal distribution that encode the conditional dependencies corresponding to three edges as non-zero entries in the precision matrix (Figure 1a). Then, to introduce confounding in the data, we simulate a sample specific term from a standard normal distribution, and add a scalar multiple of that to genes 2 through 6 (Figure 1d). Finally, to correct the data, we regress out the first principal component from the confounded data (Figure 1g). We used graphical lasso to reconstruct networks using the three versions of the data. The code for this simulation example and network reconstruction can be found at: `https://github.com/leekgroup/networks_correction/blob/master/publication_rmd/simulation_example_fig1/figure1.Rmd`

2.2 Simulation with scale-free networks

We simulated 10,000 observations from a multivariate gaussian distribution that encode conditional dependencies across 100 genes corresponding to a sale-free network. This was obtained with B-A algorithm implemented in 'huge.generator' in '*huge*' R package. Next to introduce confounding in the data, we simulated a sample specific term from a standard normal distribution, and added a scalar multiple of that to genes 20 genes in the data. To correct the data, we regressed out the first principal component from the confounded data. We used graphical lasso to reconstruct networks using the three versions of the data.
We also simulated 350 observations from a multivariate gaussian distribution that encode conditional dependencies across 5000 genes - sample and gene numbers similar to those in our empirical experiments. We simulated two sample specific terms, and two gene specific terms to introduce weighted confounding to 1500 genes multiplied by a scalar constant. This confounding data was corrected by regressing 2 PCs (as estimated by the permutation procedure). We used graphical lasso to reconstruct networks with three versions of data.
The code for these simulation examples and network reconstruction can be found at: `https://github.com/leekgroup/networks_correction/blob/master/publication_rmd/`

2.3 Determining sample specific estimate of GC bias

Studies have shown that GC content of genes have significant impact on sequencing read coverage in DNA-seq and RNA-seq experiments. This eventually introduces sample specific biases in expression quantification. To quantify the effect of GC bias, using transcript level fasta files from Gencode v25 we first computed the GC% of each transcript by:

$$GC\%(T) = \frac{(\#G + \#C)}{(\#A + \#T + \#G + \#C)}$$

We summarized GC content of genes, by averaging over all transcripts belonging to the gene. Suppose k transcripts were transcribed from gene G_i then,

$$GC\%(G_i) = \frac{\sum_{j=1}^{k} GC\%(T_j)}{k}$$

Next using a linear model, we obtain sample specific estimates of GC content of genes:

$$E_i = \mu + \beta_i \times G$$

where, E_i is the vector of expression values of all genes in sample i, G is the GC content for each gene and β_i is the estimate of GC bias for sample i.

2.4 Network reconstruction using GTEx data

Based on sample size we used gene expression RNAseq data from eight tissues in the GTEx project[Consortium et al., 2017] that included whole blood, lung, skeletal muscle, tibial artery, sun-exposed skin, tibial nerve, subcutaneous adipose, and thyroid. In each tissue we filtered for non-overlapping protein coding genes that had scaled expression (counts scaled by the total coverage

of the sample) of at least 0.1 25% of total number of observations. Next, we log2 transformed the scaled gene expression data, and performed the following steps to select the most variable 5000 genes across all tissues, correct gene expression data, and build co-expression networks.

(a) Select genes expressed in all five tissues.

(b) For each tissue, assign a rank to each gene by variance, such that the most variable gene is ranked first and least variable gene is ranked in last.

(c) Using the ranked list of genes from five tissues, assign an average rank to each gene across five tissues.

(d) Select top 5000 genes based on average rank for network inference with WGCNA and graphical lasso.

We used multiple approaches to correct gene expression data from each tissue individually as described below:

- Residuals from RIN/Exonic Rate/ GC bias: Using a linear model, we regressed the RNA integrity number (RIN), exonic rate or sample specific estimate of GC bias on the expression data and computed the residuals

- Residuals from multiple covariate correction: In each tissue individually, we estimated expression percent variance $R2$ explained by the known technical confounders. Next, using a linear model we regressed the technical covariates with $R2 \geq 0.01$ in a tissue and computed the residuals. (Supplementary Table 4)

- Residuals from principal components: For each tissue, principal component based gene expression residuals were computed as described in above.

Prior to reconstructing co-expression networks with WGCNA and graphical lasso, we transformed the uncorrected and corrected expression of each gene to a Gaussian distribution by projecting the expression of each gene to the quantiles of a standard normal. To reconstruct unsigned weighted co-expression networks with WGCNA, we identified lowest power for which scale-free fit R^2 between $\log(p(k))$ and $\log(k)$ exceeds 0.85. Here $p(k)$ is the fraction of nodes in the network with at least k neighbors. After that we used the 'blockwisemodules' function in the *WGCNA* CRAN package to perform co-expression module detection at varying cut-heights of hierarchical dendrogram ranging from 0.9 to 1.0. For networks reconstructed with WGCNA, we considered all genes in the same module to be a fully-connected subgraph.

For reconstruction of co-expression networks with graphical lasso, we first computed the gene covariance matrix and then used 'QUIC' function in the QUIC R package to infer co-expression networks with penalization parameter λ ranging from 0.3 to 1.0.

2.4.1 Network evaluation

Since the underlying network structure is generally unknown, we used a) genes known to be functional in the same pathways and b) known transcription factors and their targets as ground truth to assess these networks.

- ***Canonical pathway databases:*** We downloaded the latest pathway information (2016) from KEGG, Biocarta and Pathway Interaction Database from Enrichr [Chen et al., 2013, Kuleshov et al., 2016], that were also annotated as canonical pathways by MSigDB [Liberzon et al., 2011]. The number of pathways/genesets in each of these databases were:
  - KEGG - 293
  - Biocarta - 237
  - Reactome - 1530
  - Pathway Interaction Database - 209

Supplementary Note 3: Effect of fewer PC correction on reconstruction of co-expression networks with WGCNA and graphical lasso

Since broad trends in co-expression may sometimes reflect distant regulatory relationships between genes,to ensure that we are not removing true long range signals, we also reconstructed networks with data corrected for one quarter and half the number of PCs estimated by our correction method. With WGCNA, we found that using a half of the esimtated number of PCs sometimes performed better in lung and skin. For the remaining tissues half-PC correction does reduce false discoveries compared to uncorrected data, however using the complete number of estimated PCs performs better (Supplementary Figure 6).

With graphical lasso networks, correcting data with fewer PCs does improve FDR compared to uncorrected data. However, the networks built with data corrected with complete PCs performed either better or similar to fewer number of PCs(Supplementary Figure 7).

Supplementary Note 4: Supplementary Figures and Tables

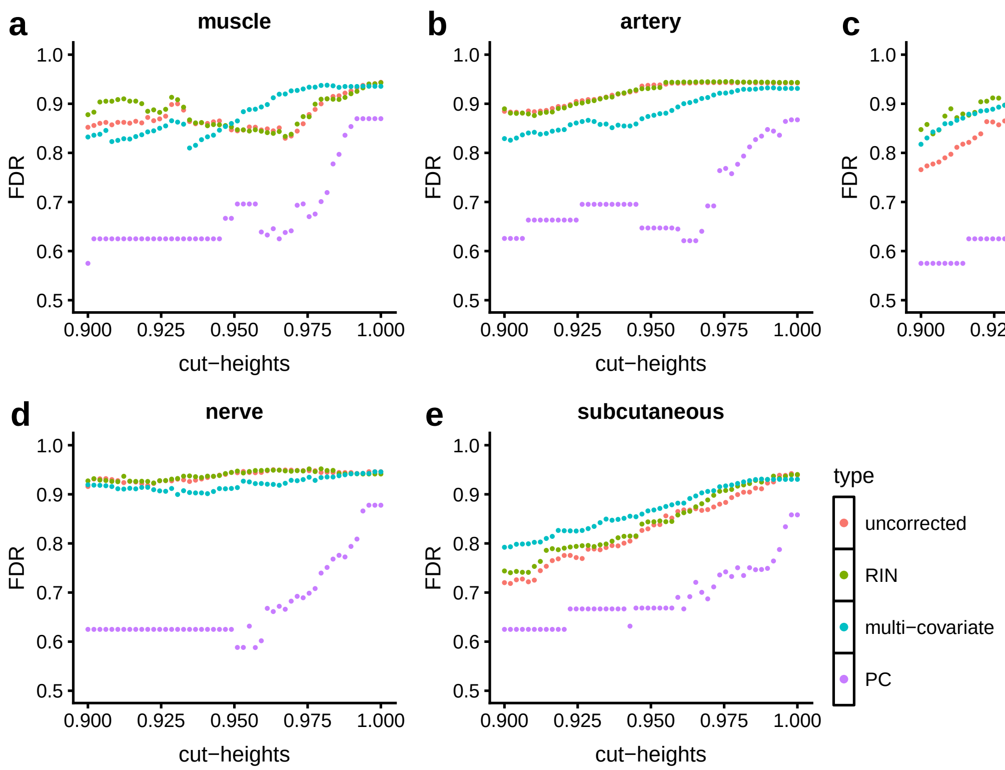

Supplementary Figure 1: False discovery rates of WGCNA networks obtained at a varying cut-heights with uncorrected, RIN corrected, multiple covariate corrected and PC corrected data. Most tissues show considerable reduction in false discoveries after PC correction. PC correction shows only moderate improvement on FDR in sun-exposed skin.

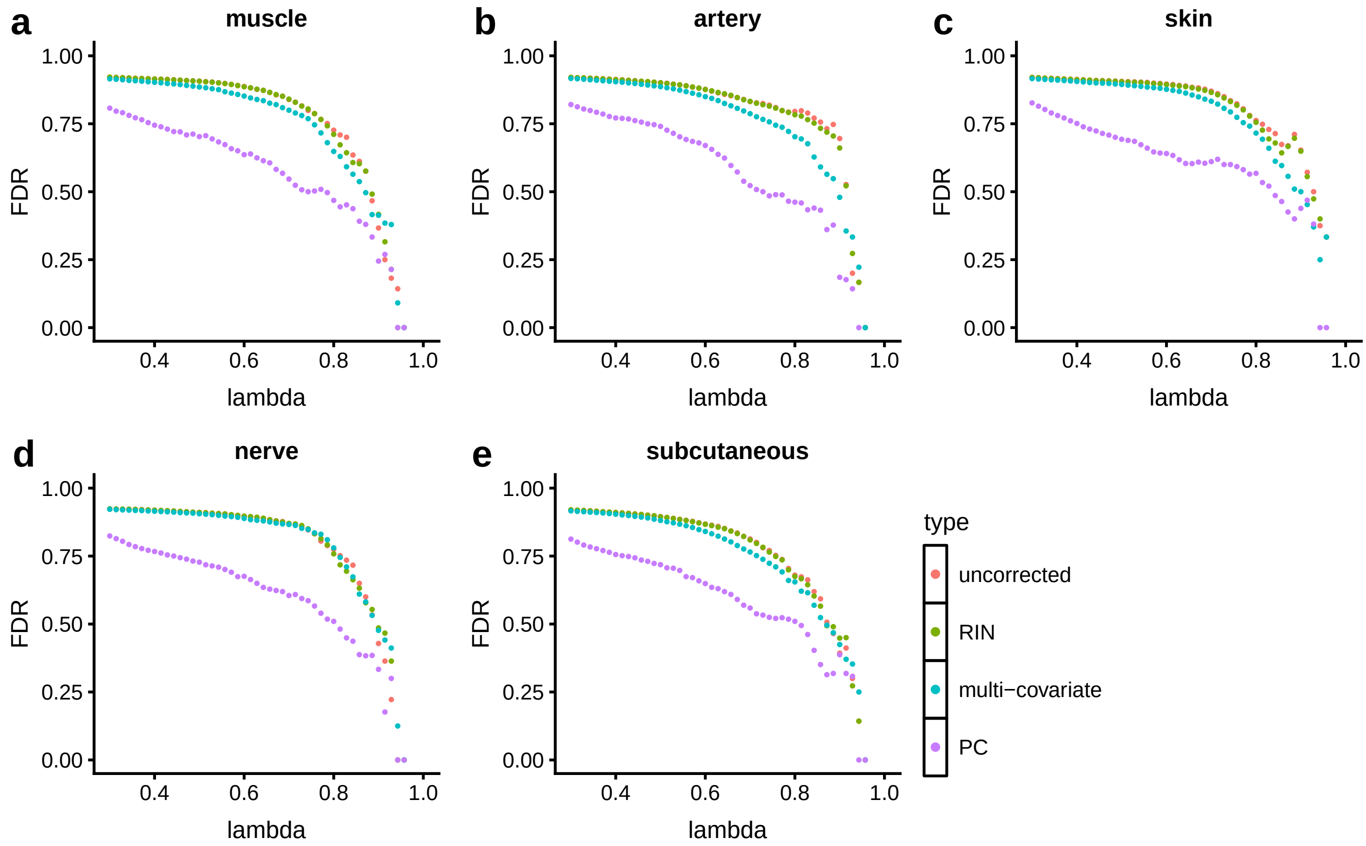

Supplementary Figure 2: False discovery rates of graphical lasso networks using canonical pathway databases. Networks were obtained at a varying values of penalty parameter (0.3 - 1.0). Each color corresponds to the correction approach, and each point corresponds to the network obtained at a specific lambda.

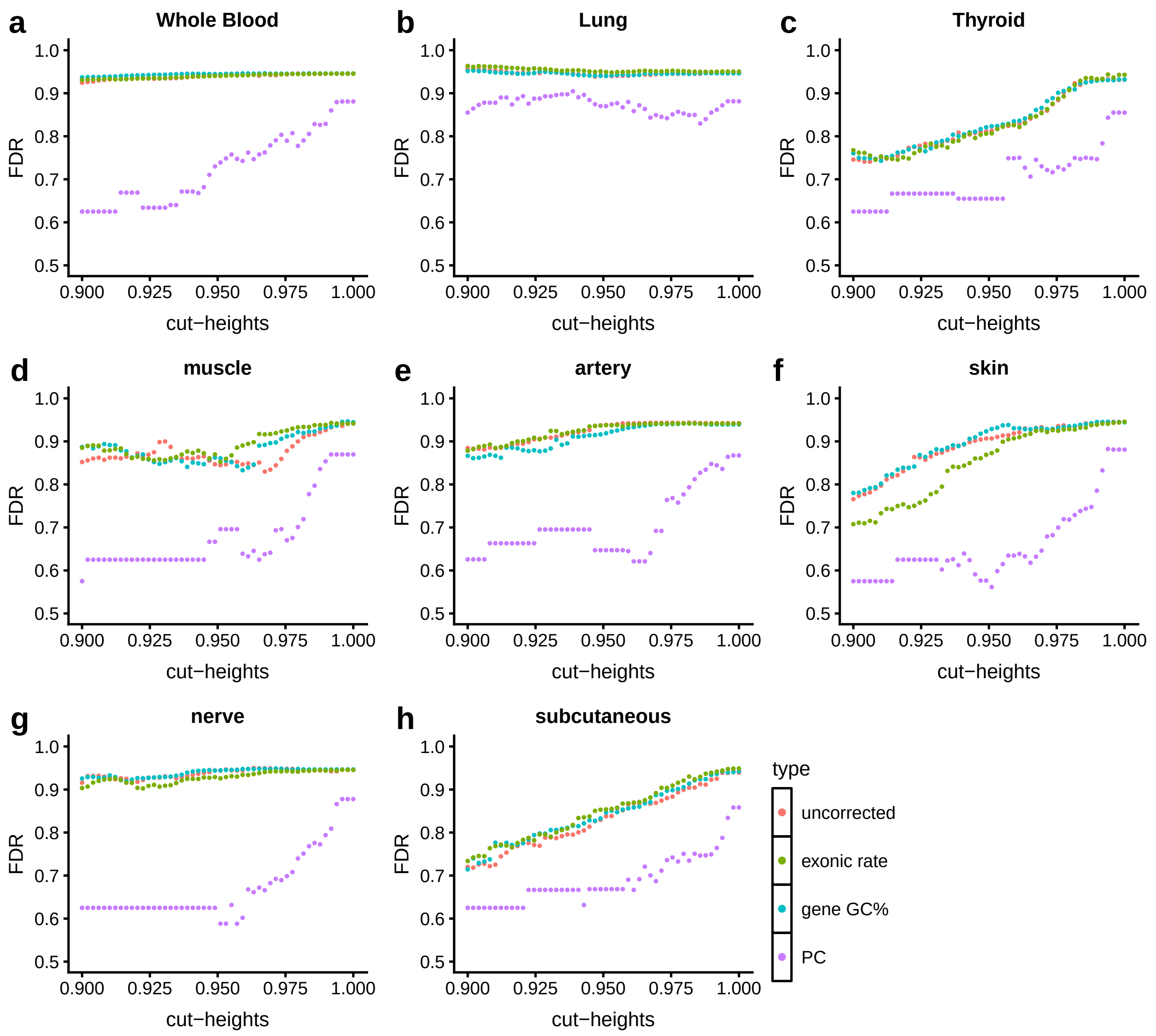

Supplementary Figure 3: False discovery rates of WGCNA modules using canonical pathway databases. Each color corresponds to the correction approach, and each point in the figure corresponds to FDR of the network at specific cut-height. Exonic rate and gene GC% are the known confounder used in this figure.

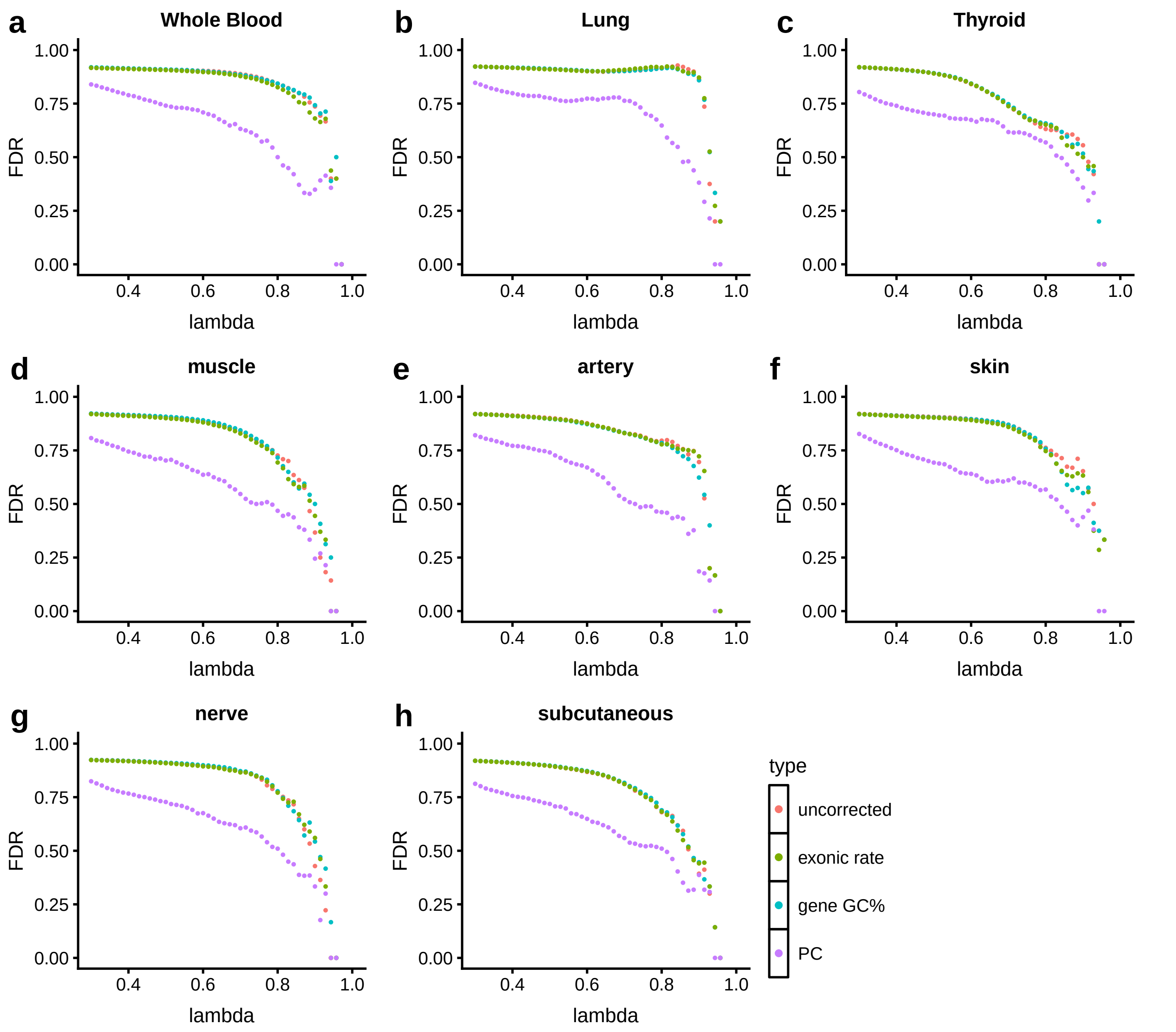

Supplementary Figure 4: False discovery rates of graphical lasso networks using canonical pathway databases. Networks were obtained at a varying values of penalty parameter (0.3 - 1.0). Each color corresponds to the correction approach, and each point corresponds to the network obtained at a specific lambda. Exonic rate and gene GC% are the known confounder used in this figure.

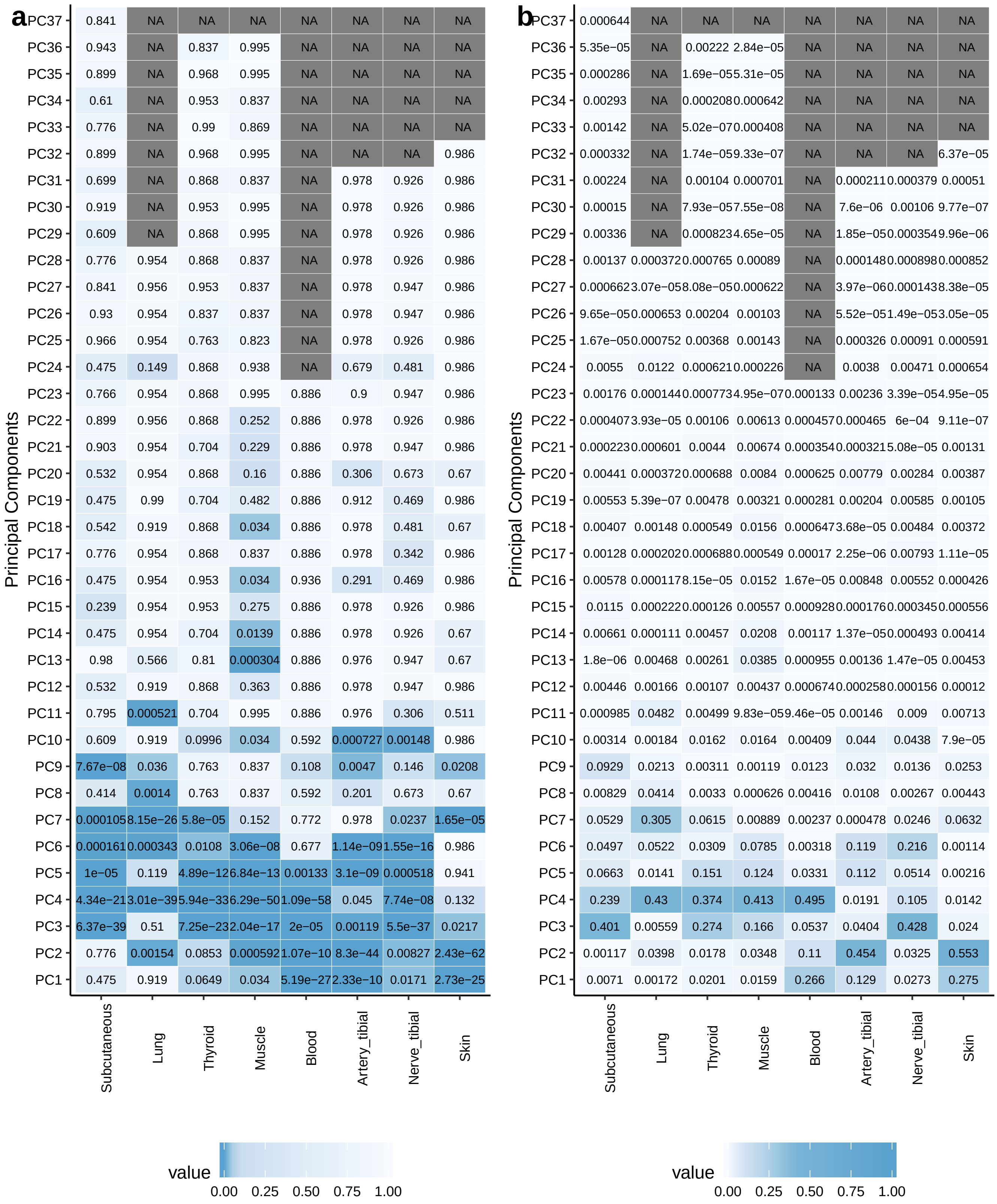

Supplementary Figure 5: Principal component loadings of gene expression are significantly associated with estimates of sample specific GC bias. Association was tested using a linear model. Panel (a) shows BH adjusted p-values and (b) shows R-squared

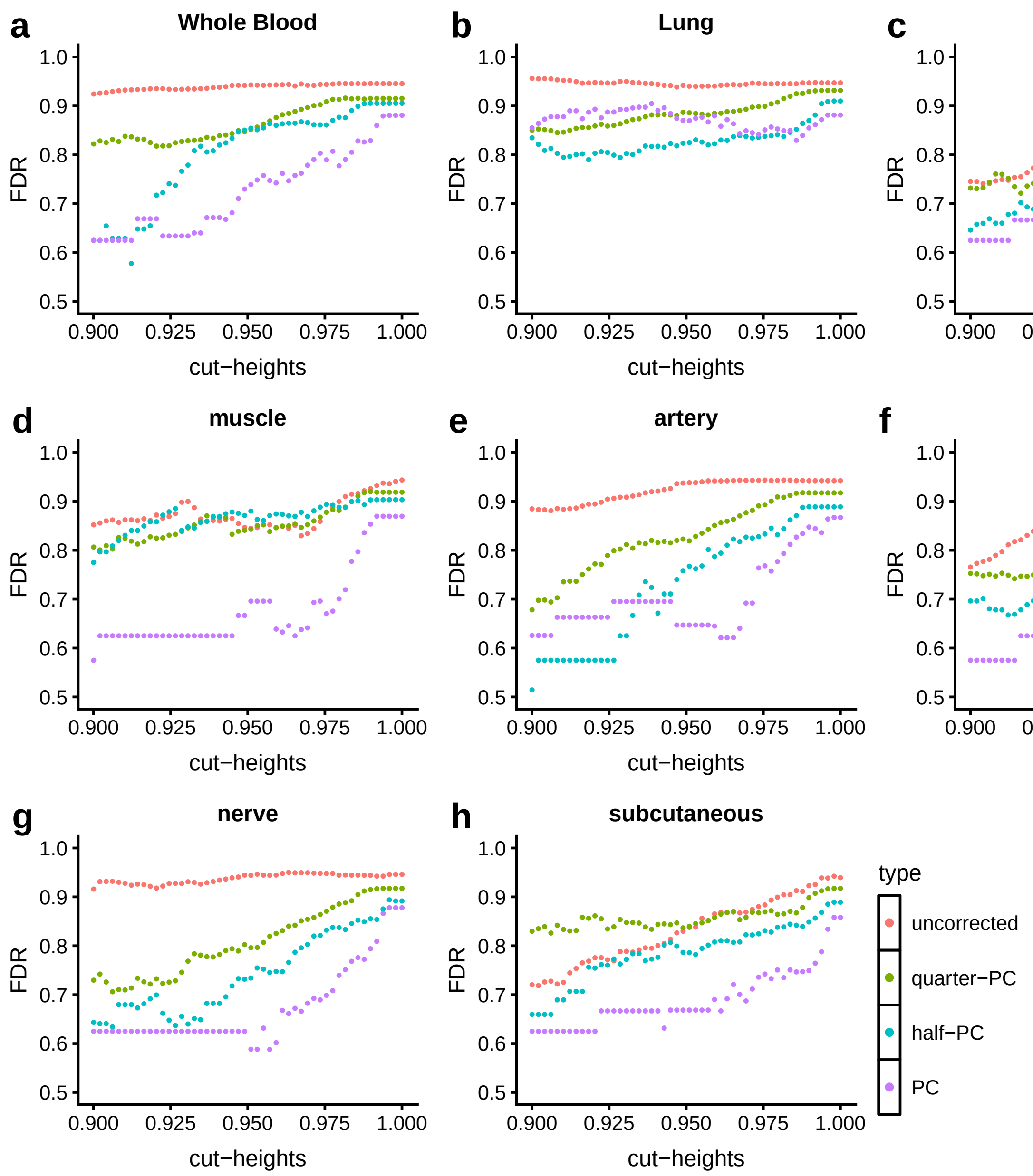

Supplementary Figure 6: Comparing false discovery rates of WGCNA modules using canonical pathway databases with data corrected with fewer PCs. In this figure, we corrected the data with a half and a quarter of the number of PCs estimated to be removed (Supplementary Table 3). Each color corresponds to the correction approach, and each point in the figure corresponds to FDR of the network at specific cut-height.

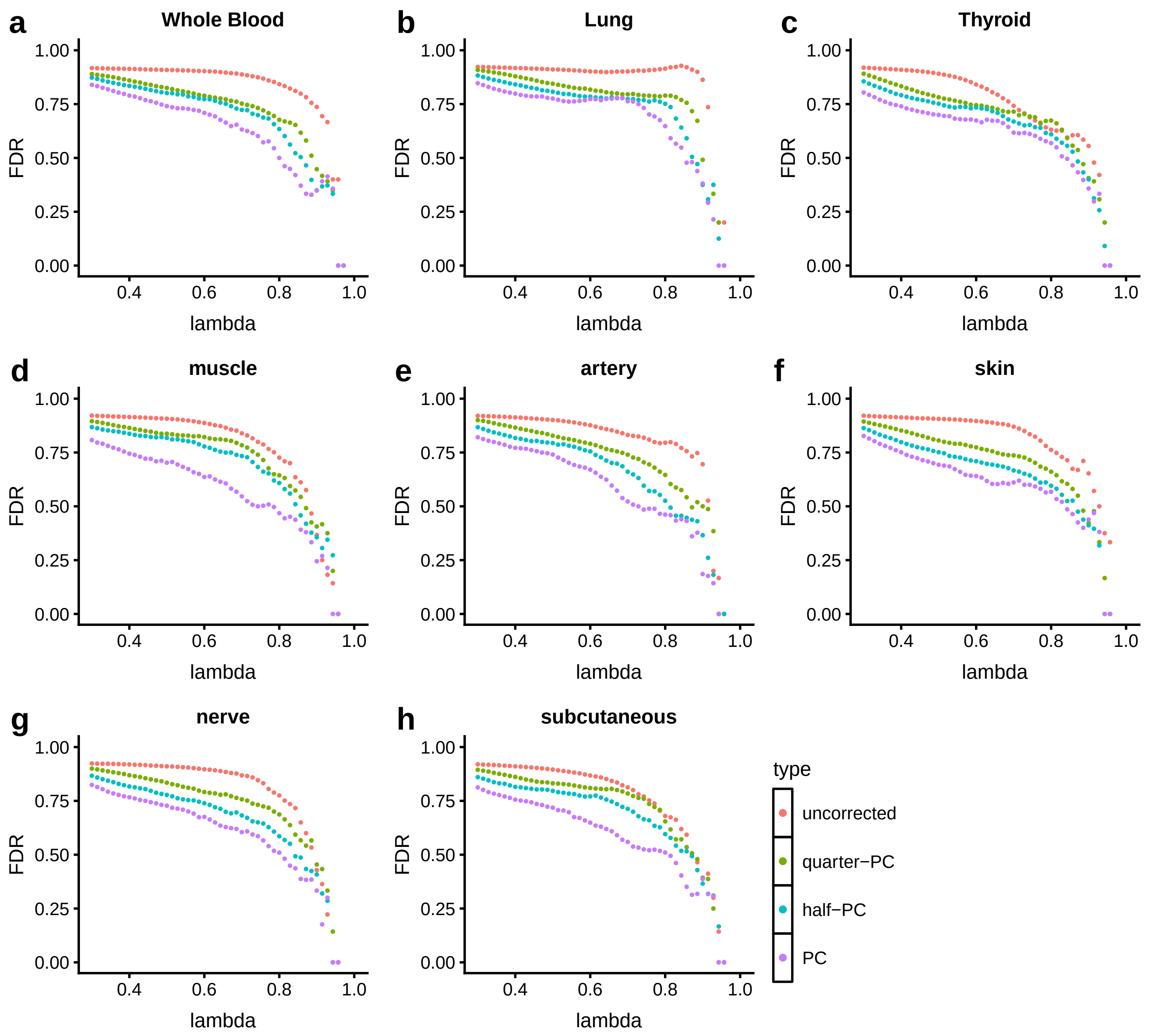

Supplementary Figure 7: Comparing false discovery rates of graphical lasso networks using canonical pathway databases with data corrected with fewer PCs. In this figure, we corrected the data with a half and a quarter of the number of PCs estimated to be removed (Supplementary Table 3). Each color corresponds to the correction approach, and each point in the figure corresponds to FDR of the network at specific value of penalty parameter (lambda = [0.3, 1.0]).

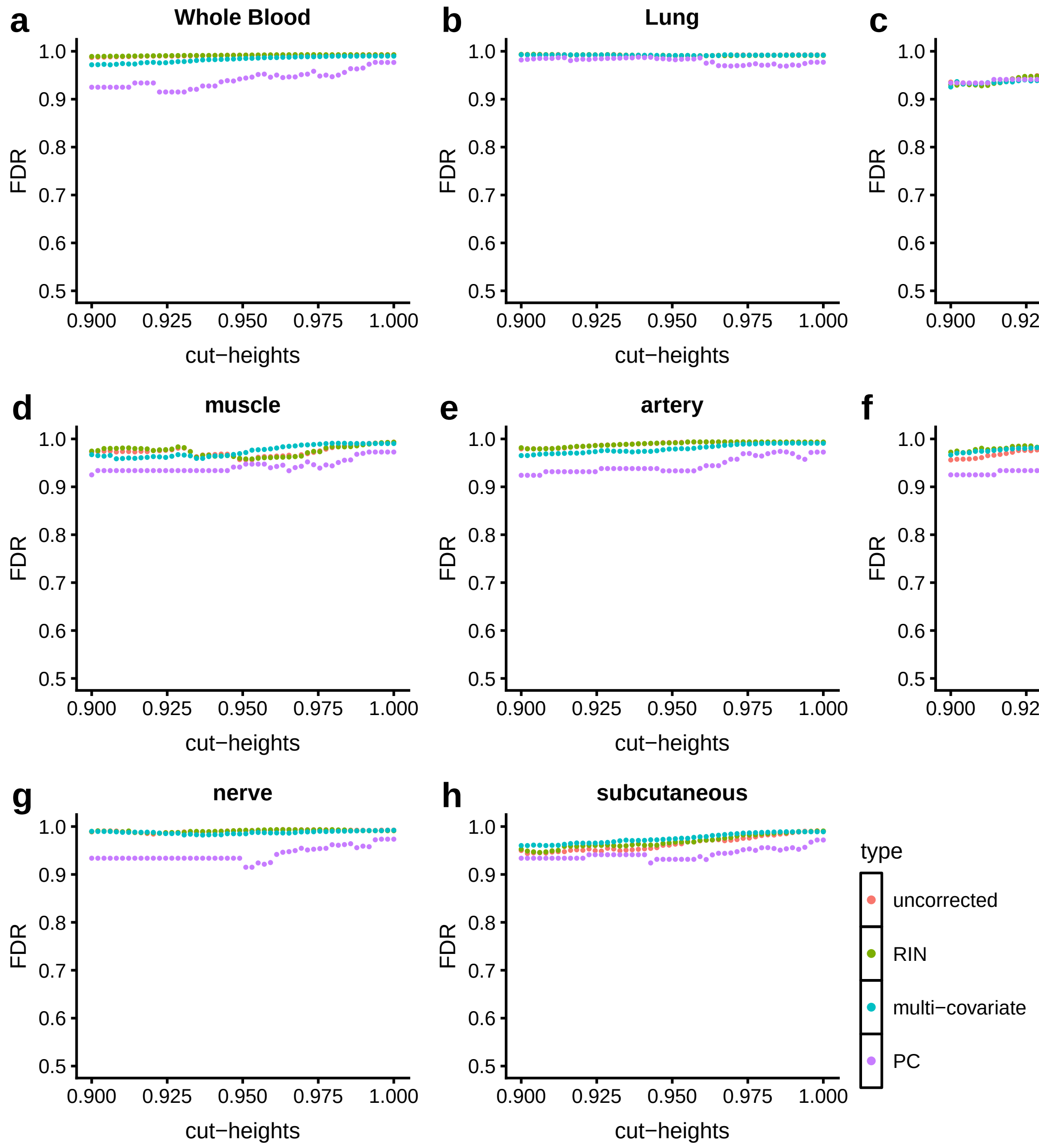

Supplementary Figure 8: False discovery rates of WGCNA networks using shared list of true positives obtained from canonical pathway database (gene pairs present in at least two pathway databases). Each color corresponds to the correction approach, and each point in the figure corresponds to FDR of the network at specific cut-height.

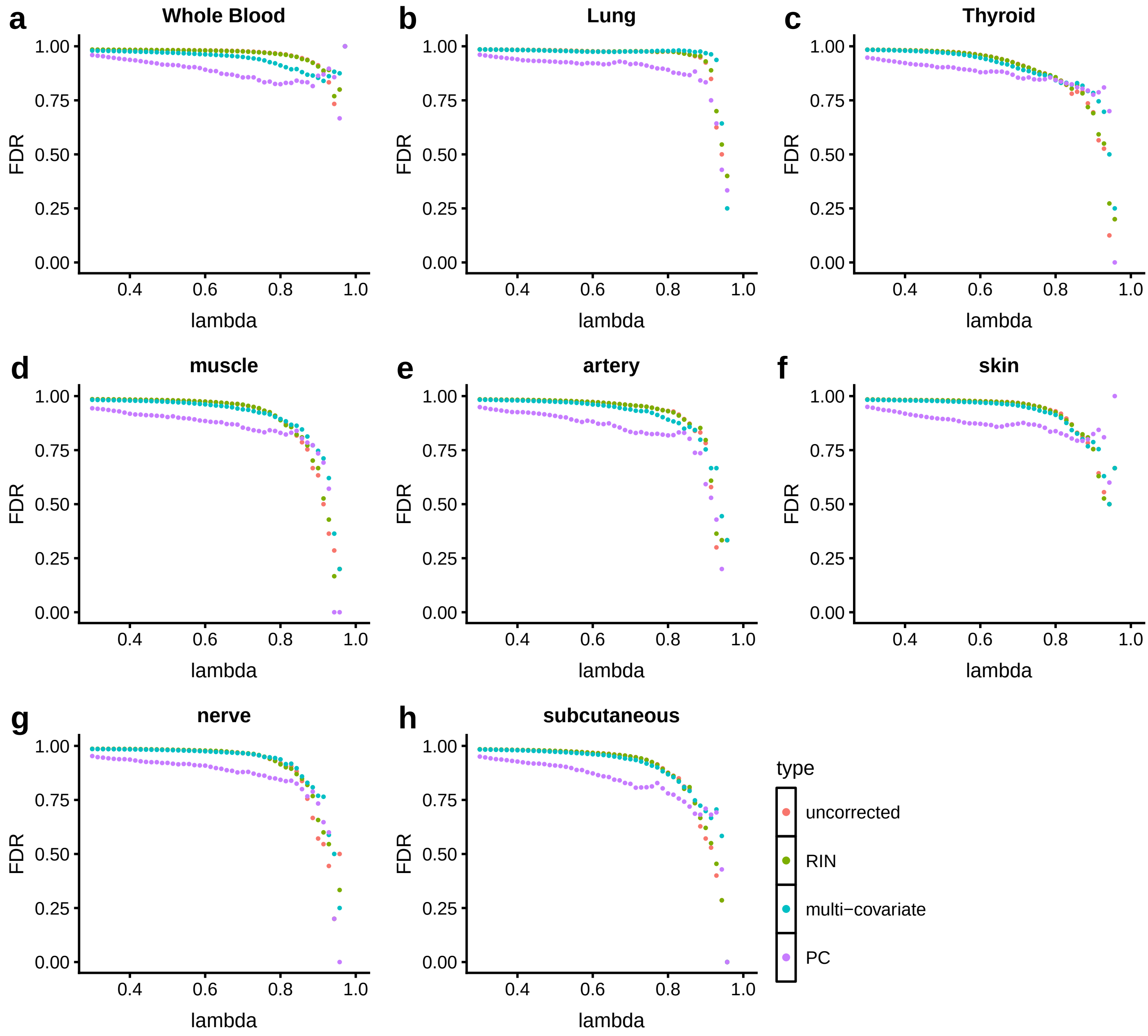

Supplementary Figure 9: False discovery rates of graphical lasso networks using shared list of true positives obtained from canonical pathway database (gene pairs present in at least two pathway databases). Each color corresponds to the correction approach, and each point in the figure corresponds to FDR of the network at specific value of penalty parameter value (lambda = [0.3, 1.0])

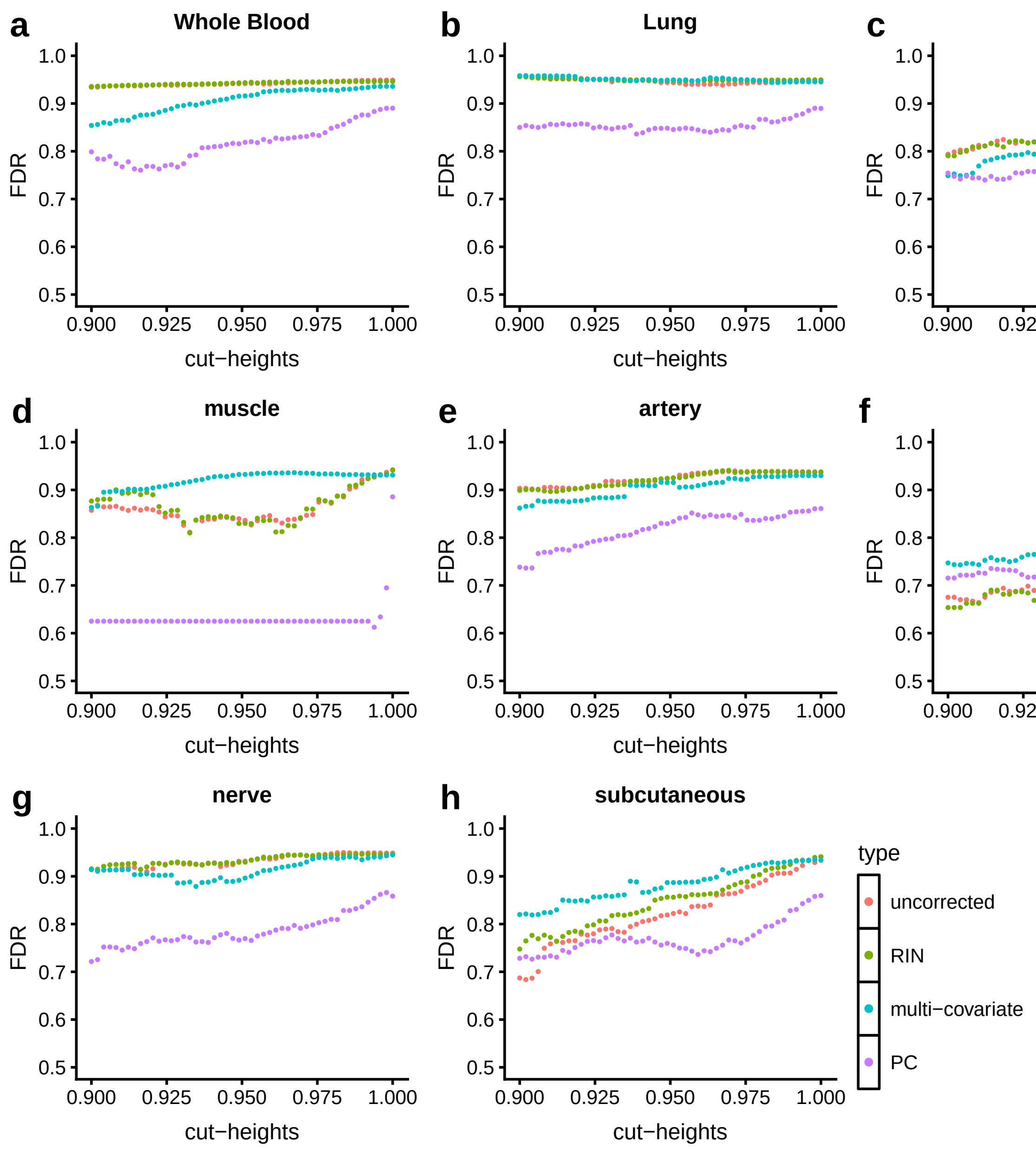

Supplementary Figure 10: False discovery rates of networks inferred with unsigned WGCNA networks using canonical pathways. Each color corresponds to the correction approach, and each point in the figure corresponds to FDR of networks obtained at varying cut-heights of hierarchical dendrogram.

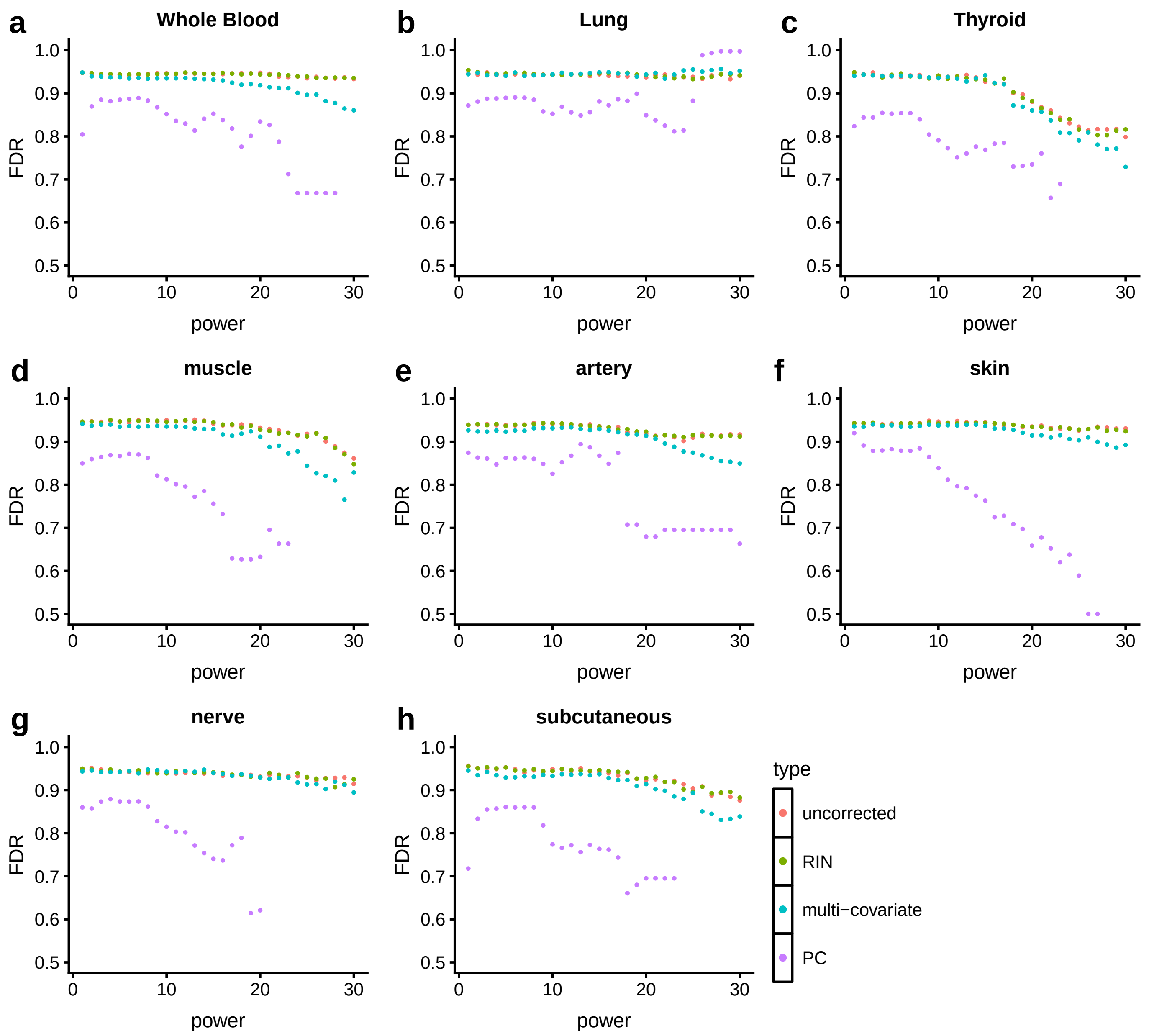

Supplementary Figure 11: False discovery rates of networks inferred with signed WGCNA networks using canonical pathways. Each color corresponds to the correction approach, and each point in the figure corresponds to FDR of networks obtained at different values of power transform β, ranging from 1 to 30.

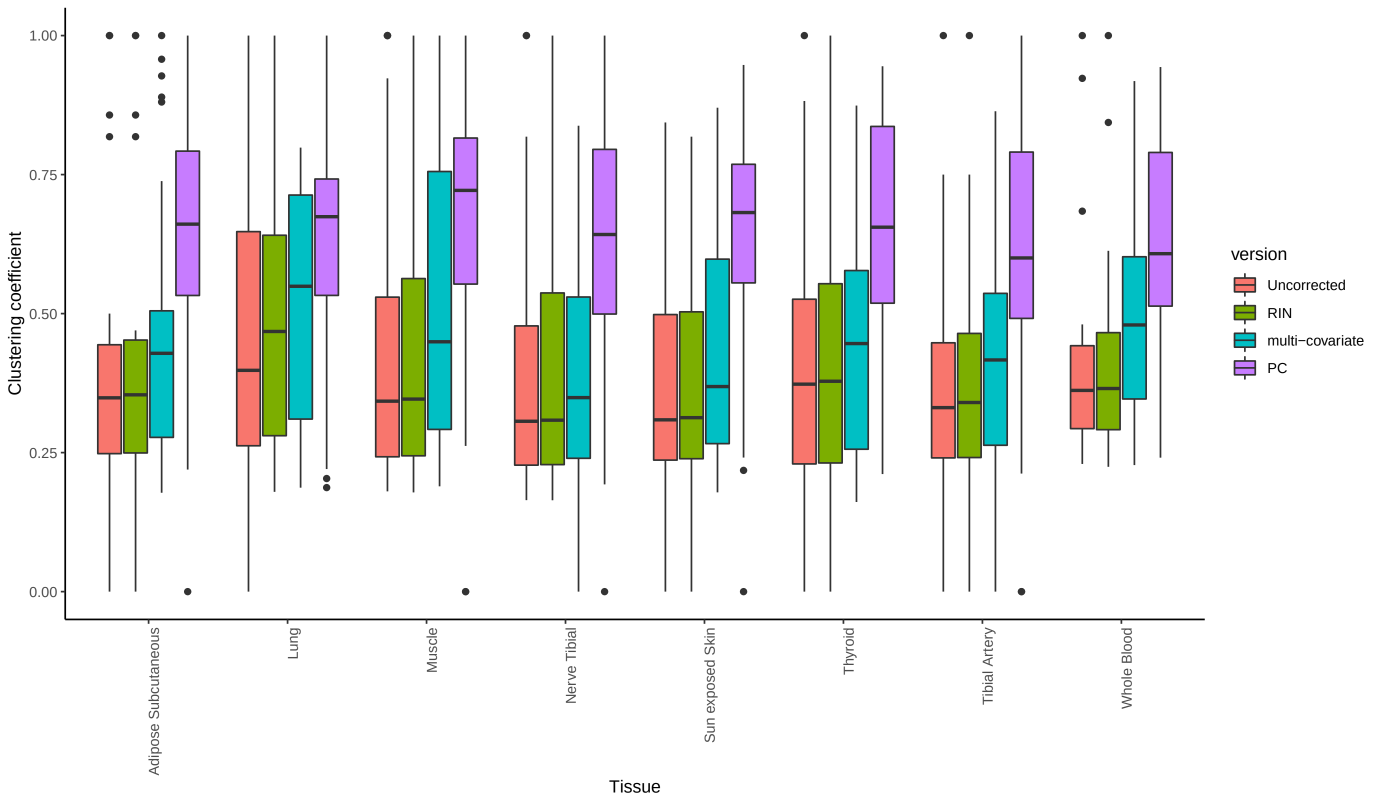

Supplementary Figure 12: Graphical lasso networks reconstructed after PC correction of gene expression measurements show higher clustering coefficient compared to uncorrected networks across all tissues. Both scale-free and small-world networks have high clustering coefficient.

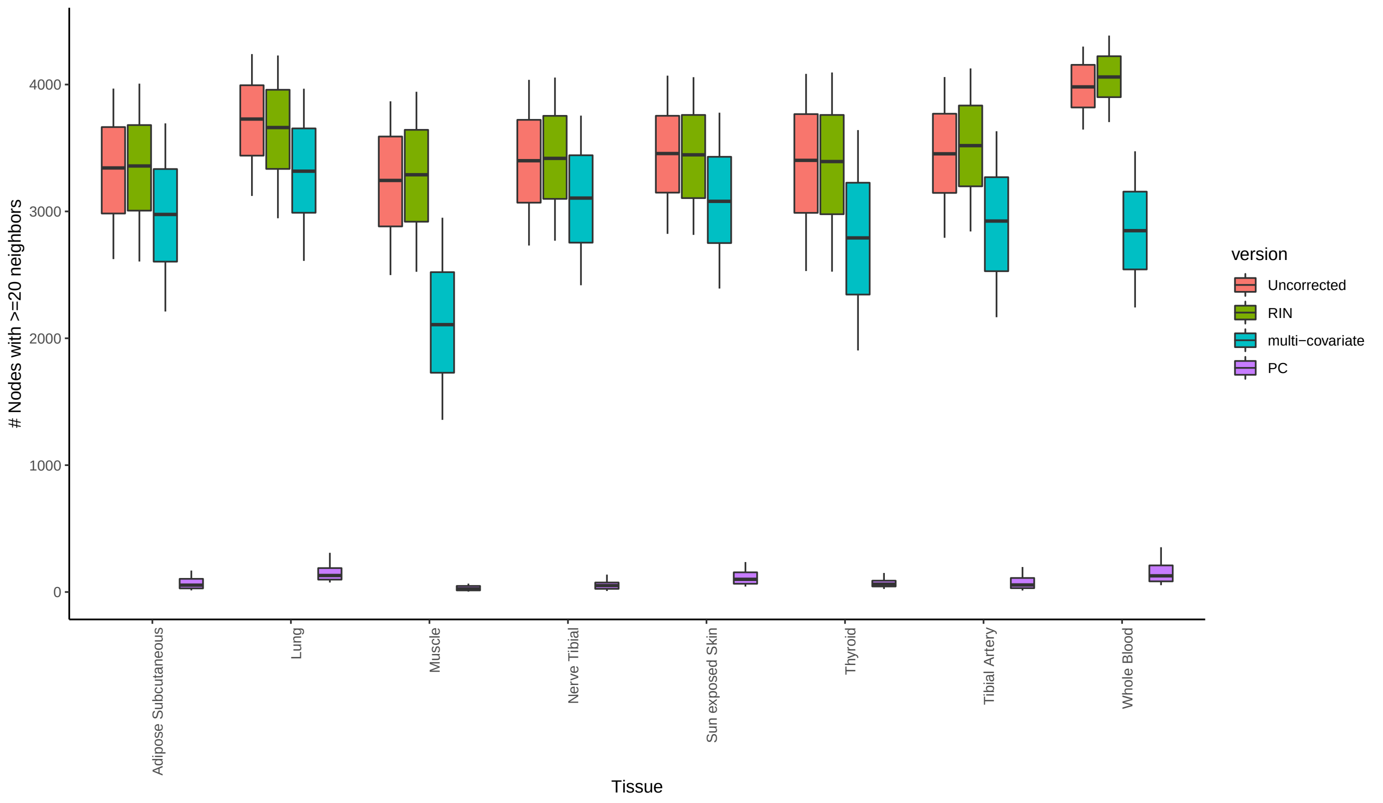

Supplementary Figure 13: Graphical lasso networks ($\lambda = [0.3, 0.43]$) reconstructed after PC correction of gene expression measurements show considerably fewer hub nodes compared to uncorrected networks across all tissues. Scale-free networks have few hub nodes

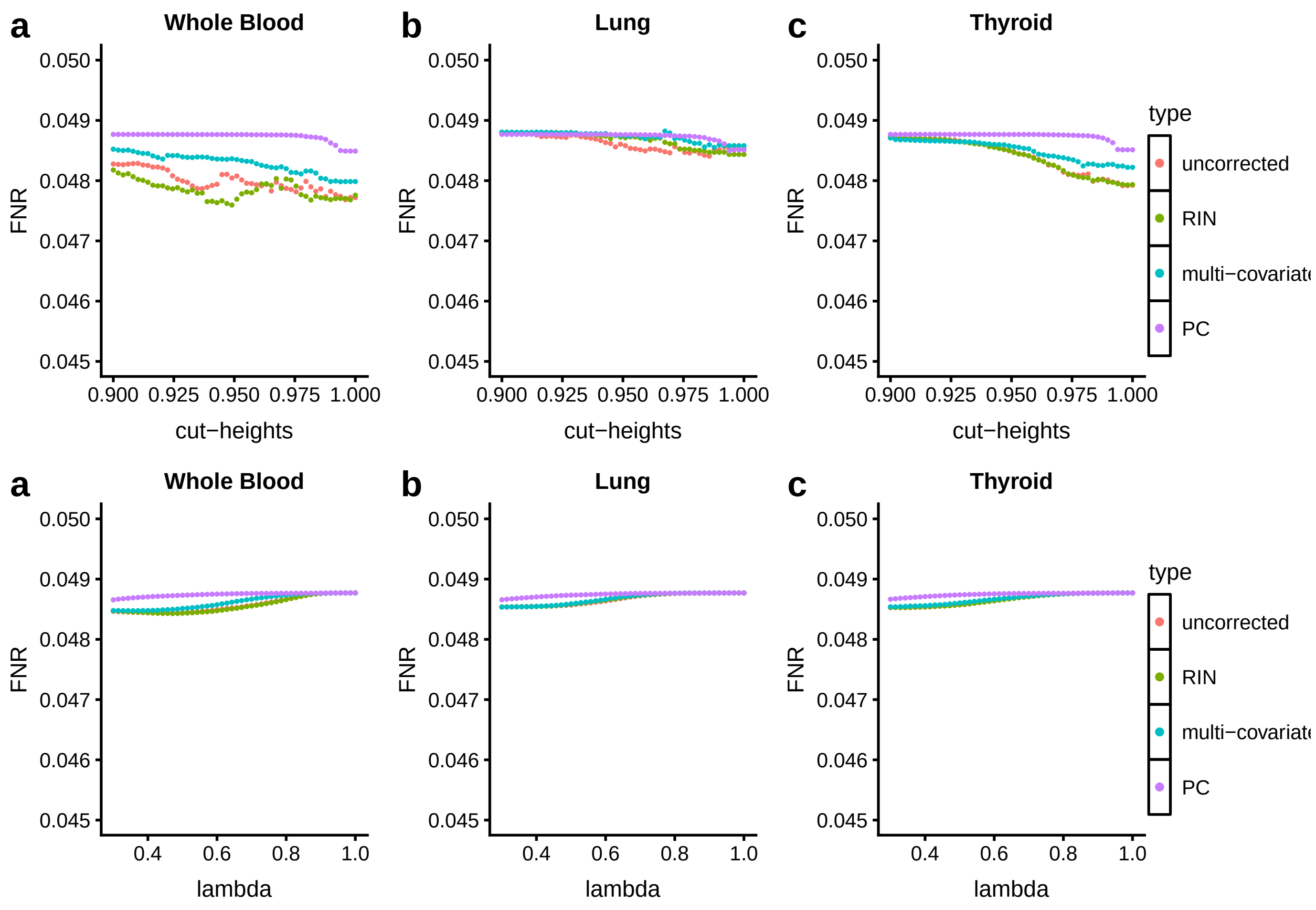

Supplementary Figure 14: Graphical lasso networks reconstructed before and after PC correction of gene expression measurements show no improvement on false negative rates.

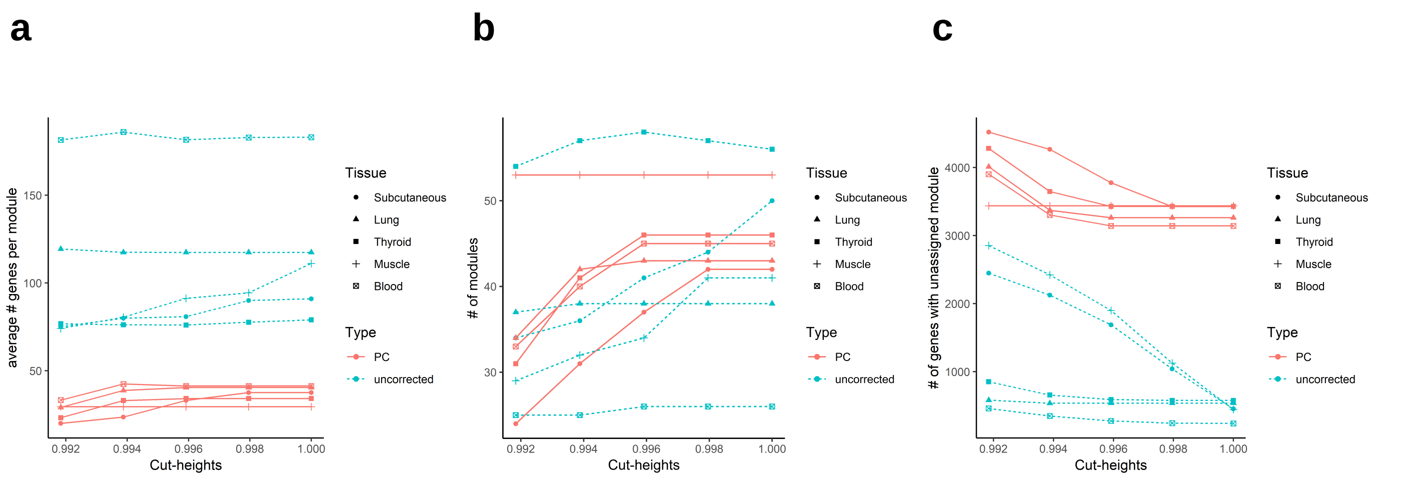

Supplementary Figure 15: Module properties of WGCNA before and after PC correction of gene expression measurements. a) On average the number of genes per module are considerably smaller in WGCNA after PC correction of data b) The number of modules identified are different and varies across tissues. The pattern was inconclusive among PC corrected and uncorrected networks. c) The number of genes assigned to gray module is considerably higher upon PC correction.

Study	Network re-construction method	Correction approach
[Zhang et al., 2013]	WGCNA	known technical factors - RIN, pH, PMI, age, batch, preservation, and gender
[Yang et al., 2014]	WGCNA	none
[Kogelman et al., 2014]	WGCNA	none, voom normalization
[Xue et al., 2014]	WGCNA	none, quantile normalization
[Miller et al., 2014]	WGCNA	none, quantile normalization
[Hawrylycz et al., 2015]	WGCNA	batch correction
[Breen et al., 2015]	WGCNA	none prior to network reconstruction. After networks were reconstructed, tested for confounding through module eigengene-trait correlations. however these did not include technical confounders like batch, etc.
[Bailey et al., 2016]	WGCNA	none, tmm normalization
[Gao et al., 2016]	Bayesian biclustering	network learning method jointly models hidden confounders
[Fromer et al., 2016]	WGCNA	known technical covariates: diagnosis status, Age of death, sex, PMI, pH, RIN, clustered processing batch, and ancestry markers
[Saha et al., 2017]	Graphical lasso	hidden factor correction
[Hoadley et al., 2018]	WGCNA	batch correction
[Lombardo et al., 2018]	WGCNA	none, quantile normalization

Supplementary Table 1: Few studies applying re-construction of co-expression networks

	# of samples
Whole Blood	393
Lung	320
Skeletal Muscle	430
Tibial Artery	332
Sun-exposed skin	356
Tibial Nerve	304
Adipose Subcutaneous	349
Thyroid	323

Supplementary Table 2: Tissue sample size

	Expression variance explained by known artifacts
Whole Blood	0.5405311
Lung	0.2486490
Skeletal Muscle	0.2723508
Tibial Artery	0.2730153
Sun-exposed skin	0.1971445
Tibial Nerve	0.1999944
Adipose Subcutaneous	0.1958540
Thyroid	0.2088201

Supplementary Table 3: Gene expression variance explained (Adjusted R^2) by measured known technical artifacts

	Total # of PCs removed
Whole Blood	23
Lung	28
Skeletal Muscle	36
Tibial Artery	31
Sun-exposed skin	32
Tibial Nerve	31
Adipose Subcutaneous	37
Thyroid	36

Supplementary Table 4: Number of principal components removed in each tissue

Tissue	Known covariate
Adipose Subcutaneous	- Code for BSS collection site<br>- RNA integrity number (RIN)<br>- Type of nucleic acid isolation batch<br>- Estimated library size<br>- Mean coefficient of variance<br>- Transcripts detected<br>- Intronic rate<br>- Expression profiling efficiency<br>- # transcripts that have at least one read in their 5' end<br>- % intragenic End 2 reads sequenced in sense direction<br>- gene GC%
Lung	- Autolysis score<br>- Code for BSS collection site<br>- RNA integrity number (RIN)<br>- Type of nucleic acid batch

	- End 2 mapping rate<br>- 3’ 50-base normalization<br>- Transcripts detected<br>- Gap percentage<br>- Intronic rate<br>- % intragenic End 1 reads sequenced in sense direction<br>- % intragenic End 2 reads sequenced in sense direction<br><br>- Gene GC%
Skeletal Muscle	- Code for BSS collection site<br>- Type of nucleic acid isolation batch<br>- chimeric pairs<br>- 3’ 50-base normalization<br>- Library size<br>- Intergenic rate<br>- Transcripts detected<br>- Gap percentage<br>- Intronic rate<br>- Mapped unique rate of total<br>- % intragenic End 1 reads sequenced in sense direction<br>- # transcripts that have at least one read in their 5’ end<br>- Duplication rate of mapped<br>- Gene GC%
Thyroid	- Code for BSS collection site<br>- Autolysis score<br>- Type of nucleic acid isolation batch<br>- RNA integrity number<br>- 3’ 50 base normalization<br>- Library size<br>- Intergenic rate<br>- Reads designated as failed by sequencer<br>- Transcripts detected<br>- Intronic rate<br>- Expression profiling efficiency<br>- # transcripts that have at least one read in their 5’ end<br>- Duplication rate of mapped<br>- % intragenic end 2 reads sequenced in sense direction<br>- Gene GC%
Whole Blood	- Mapped read count<br>- Code for BSS collection site<br>- RNA integrity number (RIN)<br>- Time point reference for Start and End times of sample procurement<br>- Chimeric pairs

	- 5' 50-base normalization<br>- 3' 50-base normalization<br>- mean coverage per base<br>- Library size<br>- Reads designated as failed by sequencer<br>- Mean coefficient of variance<br>- Transcripts detected<br>- Gap percentage<br>- Intronic rate<br>- Alternative alignments<br>- % intragenic end 2 reads sequenced in sense direction<br>- Gene GC%

Supplementary Table 5: Known covariates regressed from gene-expression data for multiple covariate based correction. The expression variance explained (adjusted R^2) by these covariates was $>= 0.01$

References

[Bailey et al., 2016] Bailey, P., Chang, D. K., Nones, K., Johns, A. L., Patch, A.-M., Gingras, M.-C., Miller, D. K., Christ, A. N., Bruxner, T. J., Quinn, M. C., et al. (2016). Genomic analyses identify molecular subtypes of pancreatic cancer. *Nature*, 531(7592):47.

[Barabási et al., 2000] Barabási, A.-L., Albert, R., and Jeong, H. (2000). Scale-free characteristics of random networks: the topology of the world-wide web. *Physica A: Statistical Mechanics and its Applications*, 281(1):69–77.

[Bhan et al., 2002] Bhan, A., Galas, D. J., and Dewey, T. G. (2002). A duplication growth model of gene expression networks. *Bioinformatics*, 18(11):1486–1493.

[Breen et al., 2015] Breen, M. S., Maihofer, A. X., Glatt, S. J., Tylee, D. S., Chandler, S. D., Tsuang, M. T., Risbrough, V. B., Baker, D. G., O'Connor, D. T., Nievergelt, C. M., et al. (2015). Gene networks specific for innate immunity define post-traumatic stress disorder. *Molecular psychiatry*, 20(12):1538.

[Carlson et al., 2006] Carlson, M. R., Zhang, B., Fang, Z., Mischel, P. S., Horvath, S., and Nelson, S. F. (2006). Gene connectivity, function, and sequence conservation: predictions from modular yeast co-expression networks. *BMC genomics*, 7(1):1.

[Chen et al., 2013] Chen, E. Y., Tan, C. M., Kou, Y., Duan, Q., Wang, Z., Meirelles, G. V., Clark, N. R., and Ma'ayan, A. (2013). Enrichr: interactive and collaborative html5 gene list enrichment analysis tool. *BMC bioinformatics*, 14(1):128.

[Consortium et al., 2017] Consortium, G., analysts:, L., Laboratory, D. A. . C. C. L., program management:, N., collection:, B., Pathology:, eQTL manuscript working group:, Battle, A.,

Brown, C. D., Engelhardt, B. E., and Montgomery, S. B. (2017). Genetic effects on gene expression across human tissues. *Nature*, 550(7675):204–213.

[Fromer et al., 2016] Fromer, M., Roussos, P., Sieberts, S. K., Johnson, J. S., Kavanagh, D. H., Perumal, T. M., Ruderfer, D. M., Oh, E. C., Topol, A., Shah, H. R., et al. (2016). Gene expression elucidates functional impact of polygenic risk for schizophrenia. *Nature neuroscience*, 19(11):1442.

[Gao et al., 2016] Gao, C., McDowell, I. C., Zhao, S., Brown, C. D., and Engelhardt, B. E. (2016). Context specific and differential gene co-expression networks via bayesian biclustering. *PLOS Computational Biology*, 12(7):1–39.

[Hawrylycz et al., 2015] Hawrylycz, M., Miller, J. A., Menon, V., Feng, D., Dolbeare, T., Guillozet-Bongaarts, A. L., Jegga, A. G., Aronow, B. J., Lee, C.-K., Bernard, A., et al. (2015). Canonical genetic signatures of the adult human brain. *Nature neuroscience*, 18(12):1832.

[Hoadley et al., 2018] Hoadley, K. A., Yau, C., Hinoue, T., Wolf, D. M., Lazar, A. J., Drill, E., Shen, R., Taylor, A. M., Cherniack, A. D., Thorsson, V., et al. (2018). Cell-of-origin patterns dominate the molecular classification of 10,000 tumors from 33 types of cancer. *Cell*, 173(2):291–304.

[Jordan et al., 2004] Jordan, I. K., Mariño-Ramírez, L., Wolf, Y. I., and Koonin, E. V. (2004). Conservation and coevolution in the scale-free human gene coexpression network. *Molecular biology and evolution*, 21(11):2058–2070.

[Kim et al., 2001] Kim, S. K., Lund, J., Kiraly, M., Duke, K., Jiang, M., Stuart, J. M., Eizinger, A., Wylie, B. N., and Davidson, G. S. (2001). A gene expression map for caenorhabditis elegans. *Science*, 293(5537):2087–2092.

[Kogelman et al., 2014] Kogelman, L. J., Cirera, S., Zhernakova, D. V., Fredholm, M., Franke, L., and Kadarmideen, H. N. (2014). Identification of co-expression gene networks, regulatory genes and pathways for obesity based on adipose tissue rna sequencing in a porcine model. *BMC medical genomics*, 7(1):57.

[Kuleshov et al., 2016] Kuleshov, M. V., Jones, M. R., Rouillard, A. D., Fernandez, N. F., Duan, Q., Wang, Z., Koplev, S., Jenkins, S. L., Jagodnik, K. M., Lachmann, A., et al. (2016). Enrichr: a comprehensive gene set enrichment analysis web server 2016 update. *Nucleic acids research*, 44(W1):W90–W97.

[Leek, 2011] Leek, J. T. (2011). Asymptotic conditional singular value decomposition for high-dimensional genomic data. *Biometrics*, 67(2):344–352.

[Liberzon et al., 2011] Liberzon, A., Subramanian, A., Pinchback, R., Thorvaldsdóttir, H., Tamayo, P., and Mesirov, J. P. (2011). Molecular signatures database (msigdb) 3.0. *Bioinformatics*, 27(12):1739–1740.

[Lombardo et al., 2018] Lombardo, M. V., Moon, H. M., Su, J., Palmer, T. D., Courchesne, E., and Pramparo, T. (2018). Maternal immune activation dysregulation of the fetal brain transcriptome and relevance to the pathophysiology of autism spectrum disorder. *Molecular psychiatry*, 23(4):1001.

[Miller et al., 2014] Miller, J. A., Ding, S.-L., Sunkin, S. M., Smith, K. A., Ng, L., Szafer, A., Ebbert, A., Riley, Z. L., Royall, J. J., Aiona, K., et al. (2014). Transcriptional landscape of the prenatal human brain. *Nature*, 508(7495):199.

[Newman, 2003] Newman, M. E. (2003). The structure and function of complex networks. *SIAM review*, 45(2):167–256.

[Rzhetsky and Gomez, 2001] Rzhetsky, A. and Gomez, S. M. (2001). Birth of scale-free molecular networks and the number of distinct dna and protein domains per genome. *Bioinformatics*, 17(10):988–996.

[Saha et al., 2017] Saha, A., Kim, Y., Gewirtz, A. D., Jo, B., Gao, C., McDowell, I. C., Engelhardt, B. E., Battle, A., Aguet, F., Ardlie, K. G., et al. (2017). Co-expression networks reveal the tissue-specific regulation of transcription and splicing. *Genome research*, 27(11):1843–1858.

[Van Noort et al., 2004] Van Noort, V., Snel, B., and Huynen, M. A. (2004). The yeast coexpression network has a small-world, scale-free architecture and can be explained by a simple model. *EMBO reports*, 5(3):280–284.

[Xue et al., 2014] Xue, J., Schmidt, S. V., Sander, J., Draffehn, A., Krebs, W., Quester, I., De Nardo, D., Gohel, T. D., Emde, M., Schmidleithner, L., et al. (2014). Transcriptome-based network analysis reveals a spectrum model of human macrophage activation. *Immunity*, 40(2):274–288.

[Yang et al., 2014] Yang, Y., Han, L., Yuan, Y., Li, J., Hei, N., and Liang, H. (2014). Gene co-expression network analysis reveals common system-level properties of prognostic genes across cancer types. *Nature communications*, 5:3231.

[Zhang et al., 2013] Zhang, B., Gaiteri, C., Bodea, L.-G., Wang, Z., McElwee, J., Podtelezhnikov, A. A., Zhang, C., Xie, T., Tran, L., Dobrin, R., et al. (2013). Integrated systems approach identifies genetic nodes and networks in late-onset alzheimer's disease. *Cell*, 153(3):707–720.
