# Supplementary material for: Addressing confounding artifacts in reconstruction of gene co-expression networks
Source: Genome Biol. 2019 May 16;20:94. doi: 10.1186/s13059-019-1700-9 (PMC6521369; doi:10.1186/s13059-019-1700-9)
Supplement: Supplementary file 2 — Scale-free simulation (R notebook) (HTML 772 kb) [file 13059_2019_1700_MOESM2_ESM.html]

Scale-free simulation - Large sample size


# Scale-free simulation - Large sample size

Scale-free simulation with 100 genes and 10,0000 samples

```
library(huge, quietly = T)
```

```
## Warning: package 'huge' was built under R version 3.5.2
```

```
## Warning: package 'igraph' was built under R version 3.5.2
```

```
library(sva, quietly = T)
```

```
## Warning: package 'mgcv' was built under R version 3.5.2
```

```
lambda=seq(0,1,length.out=200)

set.seed(101)
## generate simulated scale free network
dat <- huge.generator(n = 10000, d = 100, graph = "scale-free", v = NULL, u = NULL,
               g = NULL, prob = NULL, vis = F, verbose = TRUE)
```

```
## Generating data from the multivariate normal distribution with the scale-free graph structure....done.
```

```
sim.dat <- dat$data
n <- nrow(sim.dat)
p <- ncol(sim.dat)

## infer networks using simulated data
sim.net <- huge(sim.dat, lambda = lambda, method = "glasso", verbose = F)

## Count edges from inferred networks, and common edges
true_ecount <- sum(dat$theta == 1 & col(dat$theta) < row(dat$theta))
print(paste("The number of edges in the true network:", true_ecount))
```

```
## [1] "The number of edges in the true network: 99"
```

```
sim_ecount <- sum(sim.net$path[[39]] == 1 & col(dat$theta) < row(dat$theta))
print(paste("The number of edges in the inferred network", sim_ecount))
```

```
## [1] "The number of edges in the inferred network 99"
```

```
sim_true_ecount <- sum(dat$theta + sim.net$path[[39]] == 2 & col(dat$theta) < row(dat$theta))
print(paste("The number common edges in the inferred and true network", sim_true_ecount))
```

```
## [1] "The number common edges in the inferred and true network 99"
```

```
## confounded data

sim.confounded=sim.dat
set.seed(101)
grp=rnorm(n)
for(i in 10:30){
  sim.confounded[,i] = sim.confounded[,i] + 5*grp
}


## infer networks
sim.confounded.net <- huge(sim.confounded, lambda = lambda, method = "glasso", verbose = F)

## Count edges from inferred networks, and common edges
true_ecount <- sum(dat$theta == 1 & col(dat$theta) < row(dat$theta))
print(paste("The number of edges in the true network:", true_ecount))
```

```
## [1] "The number of edges in the true network: 99"
```

```
confounded_ecount <- sum(sim.confounded.net$path[[39]] == 1 & col(dat$theta) < row(dat$theta))
print(paste("The number of edges in the inferred network (confounded data): ", confounded_ecount))
```

```
## [1] "The number of edges in the inferred network (confounded data):  272"
```

```
sim_confounded_ecount <- sum(dat$theta + sim.confounded.net$path[[39]] == 2 & col(dat$theta) < row(dat$theta))
print(paste("The number common edges in the inferred (confounded) and true network:", sim_confounded_ecount))
```

```
## [1] "The number common edges in the inferred (confounded) and true network: 70"
```

```
## PC correction
mod=matrix(1,nrow=dim(sim.confounded)[1],ncol=1)
colnames(mod)="Intercept"
nsv=num.sv(t(sim.confounded),mod, method = "be")
print(paste("the number of PCs estimated to be removed:", nsv))
```

```
## [1] "the number of PCs estimated to be removed: 1"
```

```
ss=svd(scale(sim.confounded))
grp.est=ss$u[,1:nsv]
sim.corrected=lm(sim.confounded~grp.est)$residuals

#infer network
sim.corrected.net <- huge(sim.corrected, lambda = lambda, method = "glasso", verbose = F)

## Count edges from inferred networks, and common edges
true_ecount <- sum(dat$theta == 1 & col(dat$theta) < row(dat$theta))
print(paste("The number of edges in the true network:", true_ecount))
```

```
## [1] "The number of edges in the true network: 99"
```

```
corrected_ecount <- sum(sim.corrected.net$path[[39]] == 1 & col(dat$theta) < row(dat$theta))
print(paste("The number of edges in the inferred network (PC corrected data): ", corrected_ecount))
```

```
## [1] "The number of edges in the inferred network (PC corrected data):  99"
```

```
sim_corrected_ecount <- sum(dat$theta + sim.corrected.net$path[[39]] == 2 & col(dat$theta) < row(dat$theta))
print(paste("The number common edges in the inferred (PC corrected) and true network:", sim_corrected_ecount))
```

```
## [1] "The number common edges in the inferred (PC corrected) and true network: 99"
```
