# Supplementary material for: Addressing confounding artifacts in reconstruction of gene co-expression networks
Source: Genome Biol. 2019 May 16;20:94. doi: 10.1186/s13059-019-1700-9 (PMC6521369; doi:10.1186/s13059-019-1700-9)
Supplement: Supplementary file 3 — Scale-free simulation with sample and gene numbers matched to GTEx (R notebook). (HTML 763 kb) [file 13059_2019_1700_MOESM3_ESM.html]

Simulation matched to sample and gene numbers in empirical analysis with GTEx


# Simulation matched to sample and gene numbers in empirical analysis with GTEx

#### *Princy Parsana*

#### *February 8, 2019*

Here, we first simulated data from a multivariate gaussian distribution based on an underlying scale-free network structure in the precision matrix. We tried to match the number of samples to the number of samples in our empirical experiments done with GTEx data. This simulation included 5000 genes across 350 samples. We found that the number of false discoveries is much higher in confounded data. Further, this analysis shows that PC correction increased the number of true positives in the inferred networks - numbers that were similar to networks obtained from original simulated data(without confounding).

```
dat.theta <- readRDS("simulated_network.Rds")
sim.net <- readRDS("networks_data.Rds")
sim.confounded.net <- readRDS("networks_confounded.Rds")
sim.corrected.net <- readRDS("networks_corrected.Rds")
a=3
## Count the total number of edges in the network
true_ecount <- sum(dat.theta == 1 & col(dat.theta) < row(dat.theta))
confounded_ecount <- sapply(sim.confounded.net, function(x) sum(x == 1 & col(x) < row(x)))
sim_ecount <- sapply(sim.net,function(x) sum(x == 1 & col(x) < row(x)))
corrected_ecount <- sapply(sim.corrected.net, function(x) sum(x == 1 & col(x) < row(x)))
gc()
```

```
##           used (Mb) gc trigger  (Mb)  max used  (Mb)
## Ncells 1543097 82.5    2648304 141.5   2648304 141.5
## Vcells 7911513 60.4   88427380 674.7 127536104 973.1
```

```
## count the number of edges common with the simulated network
confounded_common <- sapply(sim.confounded.net, function(x,y) {sum((x+y) == 2 & col(x) < row(x))}, dat.theta)
sim_common <- sapply(sim.net, function(x,y) {sum((x+y) == 2 & col(x) < row(x))}, dat.theta)
corrected_common <- sapply(sim.corrected.net, function(x,y) {sum((x+y) == 2 & col(x) < row(x))}, dat.theta)
print(paste("The number of edges in the true network:", true_ecount))
```

```
## [1] "The number of edges in the true network: 4999"
```

```
print(paste("The number of edges in the inferred network", sim_ecount[a]))
```

```
## [1] "The number of edges in the inferred network 3272"
```

```
print(paste("The number of edges in the confounded network", confounded_ecount[a]))
```

```
## [1] "The number of edges in the confounded network 67131"
```

```
print(paste("The number of edges in the corrected network", corrected_ecount[a]))
```

```
## [1] "The number of edges in the corrected network 3403"
```

```
print(paste("The number common edges in the confounded and true network", confounded_common[a]))
```

```
## [1] "The number common edges in the confounded and true network 152"
```

```
print(paste("The number common edges in the inferred and true network", sim_common[a]))
```

```
## [1] "The number common edges in the inferred and true network 223"
```

```
print(paste("The number common edges in the inferred and true network", corrected_common[a]))
```

```
## [1] "The number common edges in the inferred and true network 223"
```

Next, we checked the proportion of edges from original simulated data networks that were also identified in PC corrected networks, and proportion of edges from original simulated data networks that were also identified in confounded networks. We find that the networks obtained from PC corrected data were more similar to those obtained from original simulated data compared to confounded data.

```
sim_corrected_common <- mapply(function(x,y){
  sum(x+y == 2 & col(x) < row(x))
}, sim.net, sim.corrected.net)
sim_corrected_common/(sim_ecount+1)
```

```
##  [1] 0.9301750 0.9190886 0.8976474 0.8984375 0.8571429 0.5714286 0.5000000
##  [8] 0.0000000 0.0000000 0.0000000 0.0000000 0.0000000 0.0000000 0.0000000
## [15] 0.0000000 0.0000000 0.0000000 0.0000000 0.0000000 0.0000000
```

```
sim_confounded_common <- mapply(function(x,y){
  sum(x+y == 2 & col(x) < row(x))
}, sim.net, sim.confounded.net)
sim_confounded_common/(sim_ecount+1)
```

```
##  [1] 0.5461777 0.5527975 0.5688970 0.6406250 0.7142857 0.5714286 0.5000000
##  [8] 0.0000000 0.0000000 0.0000000 0.0000000 0.0000000 0.0000000 0.0000000
## [15] 0.0000000 0.0000000 0.0000000 0.0000000 0.0000000 0.0000000
```

```
plot(sim_ecount, sim_common, col = "darkred", pch = "*", cex = 1.5, xlab = "# of edges", ylab = "True positives")
points(corrected_ecount, corrected_common, col = "cornflowerblue", pch = "*", cex = 1.5)
points(confounded_ecount, confounded_common, col = "darkgreen", pch = "*", cex = 1.5)
legend("topleft", c("original", "confounded", "PC corrected"), col = c("darkred", "cornflowerblue", "darkgreen"), pch = "*")
```
