# Supplementary material for: Addressing confounding artifacts in reconstruction of gene co-expression networks
Source: Genome Biol. 2019 May 16;20:94. doi: 10.1186/s13059-019-1700-9 (PMC6521369; doi:10.1186/s13059-019-1700-9)
Supplement: Supplementary file 4 — Tutorial vignette to apply PC correction prior to network reconstruction in an example dataset. (HTML 726 kb) [file 13059_2019_1700_MOESM4_ESM.html]

PC correction tutorial with example dataset


# PC correction tutorial with example dataset

This is a vignette for applying PC based residualization on gene expression data for building co-expression networks.

```
## Libraries and functions
library("sva")
```

```
## Warning: package 'mgcv' was built under R version 3.5.2
```

```
library("recount", quietly = T)
```

```
## Warning: package 'matrixStats' was built under R version 3.5.2
```

```
library("WGCNA", quietly = T)
```

```
## Warning: package 'WGCNA' was built under R version 3.5.2
```

```
## Warning: package 'dynamicTreeCut' was built under R version 3.5.2
```

```
## Warning: package 'fastcluster' was built under R version 3.5.2
```

```
## ==========================================================================
## *
## *  Package WGCNA 1.66 loaded.
## *
## *    Important note: It appears that your system supports multi-threading,
## *    but it is not enabled within WGCNA in R. 
## *    To allow multi-threading within WGCNA with all available cores, use 
## *
## *          allowWGCNAThreads()
## *
## *    within R. Use disableWGCNAThreads() to disable threading if necessary.
## *    Alternatively, set the following environment variable on your system:
## *
## *          ALLOW_WGCNA_THREADS=<number_of_processors>
## *
## *    for example 
## *
## *          ALLOW_WGCNA_THREADS=8
## *
## *    To set the environment variable in linux bash shell, type 
## *
## *           export ALLOW_WGCNA_THREADS=8
## *
## *     before running R. Other operating systems or shells will
## *     have a similar command to achieve the same aim.
## *
## ==========================================================================
```

```
q_normalize <- function(dat){
  n = nrow(dat)
  p = ncol(dat)
  rank.dat =  dat # matrix for ranking
  for (i in 1:p){
    rank.dat[,i] = rank(dat[,i])
  }
  U = rank.dat/(n+1)
  qnorm(U)
}
```

We will begin with loading a subsetted version of GTEx liver data. This subsetting was done for ease of tutorial

```
load("liver_subset_gtex.Rdata")
rse_raw <- t(rse_raw) # transpose data so that rows are samples and columns are gene expression measurements
```

### Apply PC based correction to confounded gene expression measurements

Using the permutation approach, we identify the number of top PCs that contribute to noise and confounding in the gene expression measurements. This noise if not removed, can contribute to spurious correlations between genes thereby introducing false positive connections in the network.

To correct for this, using a linear model we residualize gene expression measurements using the number of PCs esimated.

```
mod=matrix(1,nrow=dim(rse_raw)[1],ncol=1)
colnames(mod)="Intercept"
nsv=num.sv(t(rse_raw),mod, method = "be") ## num.sv requires data matrix with features(genes) in the rows and samples in the column

print(paste("Number of PCs estimated to be removed:", nsv))
```

```
## [1] "Number of PCs estimated to be removed: 14"
```

```
## PC residualization of gene expression data using sva_network. Rows correspond to samples, Columns correspond to features
exprs_corrected = sva_network(rse_raw, nsv)
```

```
## Quantile normalize the corrected gene expression measurements such that each expression of every gene follows a gaussian distribution

exprs_corrected_norm <- q_normalize(exprs_corrected)
```

### Build co-expression networks with WGCNA

Using PC adjusted expression measurements, we build co-expression networks with WGCNA.

```
## select soft-thresholding power transform for WGCNA
wgcna_soft_thr <- WGCNA::pickSoftThreshold(exprs_corrected_norm, networkType = "signed")
```

```
## Warning: executing %dopar% sequentially: no parallel backend registered
```

```
##    Power SFT.R.sq  slope truncated.R.sq mean.k. median.k. max.k.
## 1      1 0.000524  1.030          0.950 509.000  509.0000 532.00
## 2      2 0.014900  2.550          0.966 264.000  264.0000 290.00
## 3      3 0.000195 -0.183          0.931 140.000  139.0000 165.00
## 4      4 0.111000 -2.840          0.777  75.800   75.0000 102.00
## 5      5 0.383000 -3.730          0.697  42.200   41.1000  67.80
## 6      6 0.652000 -3.760          0.743  24.200   22.9000  48.40
## 7      7 0.811000 -3.580          0.830  14.400   13.1000  36.60
## 8      8 0.923000 -3.110          0.933   8.880    7.6600  28.90
## 9      9 0.931000 -2.770          0.939   5.760    4.5900  23.60
## 10    10 0.905000 -2.500          0.900   3.920    2.8500  19.80
## 11    12 0.984000 -1.850          0.989   2.110    1.1700  14.50
## 12    14 0.956000 -1.540          0.945   1.340    0.5390  11.10
## 13    16 0.894000 -1.470          0.865   0.970    0.2680  10.00
## 14    18 0.950000 -1.420          0.945   0.758    0.1430   9.32
## 15    20 0.933000 -1.410          0.928   0.623    0.0784   8.69
```

```
wgcna_power <- wgcna_soft_thr$powerEstimate
 if(is.na(wgcna_power)){
    print(paste("no power reached r-suared cut-off, now choosing max r-squared based power"))
    wgcna_power <- wgcna_soft_thr$fitIndices$Power[which(wgcna_soft_thr$fitIndices$SFT.R.sq == max(wgcna_soft_thr$fitIndices$SFT.R.sq))]
 }
```

```
wgcna_net <- blockwiseModules(exprs_corrected_norm, power = wgcna_power,
                       verbose = 3, numericLabels = T)
```

```
##  Calculating module eigengenes block-wise from all genes
##    Flagging genes and samples with too many missing values...
##     ..step 1
##  ..Working on block 1 .
##     TOM calculation: adjacency..
##     ..will not use multithreading.
##      Fraction of slow calculations: 0.000000
##     ..connectivity..
##     ..matrix multiplication (system BLAS)..
##     ..normalization..
##     ..done.
##  ....clustering..
##  ....detecting modules..
##  ....calculating module eigengenes..
##  ....checking kME in modules..
##  ..merging modules that are too close..
##      mergeCloseModules: Merging modules whose distance is less than 0.15
##        Calculating new MEs...
```

```
table(wgcna_net$colors)
```

```
## 
##   0   1   2   3   4   5   6 
## 799  91  25  23  22  20  20
```

WGCNA identifies 6 co-expression modules. It also contains 799 genes unassigned to any modules.
